# Generation and direct observation of a triplet arylnitrenium ion

**DOI:** 10.1038/s41467-022-31091-z

**Published:** 2022-06-16

**Authors:** Lili Du, Juanjuan Wang, Yunfan Qiu, Runhui Liang, Penglin Lu, Xuebo Chen, David Lee Phillips, Arthur H. Winter

**Affiliations:** 1 School of Life Sciences, Jiangsu University, 212013 Zhenjiang, P.R. China; 2 Department of Chemistry, The University of Hong Kong, Pokfulam Road, Hong Kong, P.R. China; 3 Key Laboratory of Theoretical and Computational Photochemistry of Ministry of Education, Department of Chemistry, Beijing Normal University, 100875 Beijing, P.R. China; 4 Department of Chemistry, Iowa State University, 2101d Hach Hall, Ames, IA 50011 USA

**Keywords:** Reaction mechanisms, Photochemistry, Excited states, Reaction kinetics and dynamics

## Abstract

Nitrenium ions are important reactive intermediates in both chemistry and biology. Although singlet nitrenium ions are well-characterized by direct methods, the triplet states of nitrenium ions have never been directly detected. Here, we find that the excited state of the photoprecursor partitions between heterolysis to generate the singlet nitrenium ion and intersystem crossing (ISC) followed by a spontaneous heterolysis process to generate the triplet *p*-iodophenylnitrenium ion (*np*). The triplet nitrenium ion undergoes ISC to generate the ground singlet state, which ultimately undergoes proton and electron transfer to generate a long-lived radical cation that further generates the reduced p-iodoaniline. Ab Initio calculations were performed to map out the potential energy surfaces to better understand the excited state reactivity channels show that an energetically-accessible singlet-triplet crossing lies along the *N-N* stretch coordinate and that the excited triplet state is unbound and spontaneously eliminates ammonia to generate the triplet nitrenium ion. These results give a clearer picture of the photophysical properties and reactivity of two different spin states of a phenylnitrenium ion and provide the first direct glimpse of a triplet nitrenium ion.

## Abstract (web summary)

Nitrenium ions are highly electrophilic reactive intermediates of formula R−N−R+, nitrogen analogue of carbenes. Here the authors report the detection of a triplet nitrenium ion using time-resolved spectroscopic methods and ab initio computations, allowing a glimpse at the properties and behavior of this important class of intermediates.

## Introduction

Nitrenium ions are highly reactive electron-deficient nitrogen compounds with the general formula of RNR’^+^ and the hypovalent nitrogen bears a positive charge. One of the long-standing motivations for studying nitrenium ions, especially with respect to arylnitrenium ions, is due to their chemical toxicology in some biological systems. It has been proposed that DNA damage caused by enzymatically activated aromatic amines proceeds via arylnitrenium ion intermediates that can then result in carcinogenic processes and convert healthy cells into cancer cells under certain circumstances^1–9^. Therefore, arylnitrenium ions are considered as critical intermediates in some DNA chemical damage processes.

**Fig. 1:**
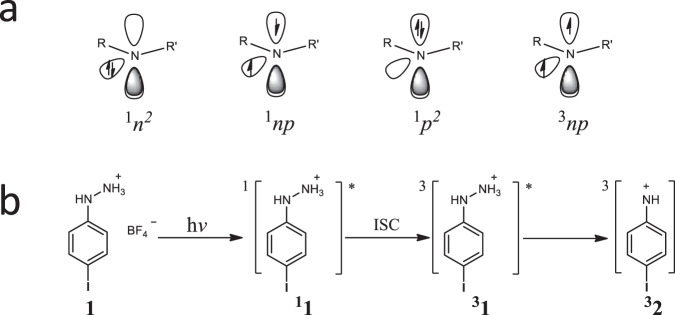
Introduction of the nitrenium ions. **a** Electronic configurations of nitrenium ions: a closed-shell singlet state (^*1*^*n*^*2*^), an open-shell singlet state (^*1*^*np*), a second closed-shell singlet state (^*1*^*p*^*2*^), and an open-shell triplet state (^*3*^*np*), (**b**) proposed mechanism for the generation of triplet phenylnitrenium ion ^**3**^**2** (*np*): after excitation, the singlet excited state of ^1^**1** could go through the intersystem crossing (ISC) process to produce the triplet excited state of ^3^**1**, which could generate the triplet nitrenium ion ^3^**2** through the N–N bond cleavage.

Nitrenium ions are isoelectronic with carbenes. Considering just the orbitals and electrons on the hypovalent nitrogen, they have four electronic configurations (Fig. 1a), including a closed-shell (*n*^*2*^) singlet state, an open-shell (*np*) singlet state, a second closed-shell (*p*^*2*^) singlet state and an open-shell (*np*) triplet state. Most of the arylnitrenium ions observed have a *n*^*2*^ singlet ground state with the contribution of electron density from the filled π orbital of the phenyl ring into the vacant *p* orbital at the nitrenium center^10–12^. Except for the parent nitrenium ion, NH_2_^+^, which is a triplet ground state, few studies have been reported to predict the existence of other triplet nitrenium ions. Gassman and Cryberg^13^ found that the hydrogen abstraction product was isolated after methanolysis of 4,7,7-trimethyl-2-chloro-2-azabicyclo[2.2.1]heptane, which indicated the existence of the triplet nitrenium ion. Falvey and coworkers revealed that a series of *N-tert*-butyl-*N*-(2-acetyl-4-substitued) phenylnitrenium ions appeared to have a triplet ground state by utilizing trapping studies with the presence of H donors to form the parent amine, although the nitrenium ion was not directly detected in these studies^14–16^. Moreover, by computing the singlet-triplet gap, some substituted nitrenium ions have been identified to be stabilized triplet nitrenium ions^12,17–20^. However, the reactivity of triplet nitrenium ions is inferred, as there are no reports of the direct observation of triplet nitrenium ions by laser flash photolysis in solution.

To directly observe a triplet nitrenium ion, we considered that appending a heavy iodine atom on the photoprecursor would accelerate intersystem crossing (ISC), since heavy atoms were known to enhance the probability of singlet to triplet conversion through coupling of spin and orbital angular momenta^21^. The triplet excited state of the photoprecursor, 2-(4-iodo-phenyl)hydrazin-1-ium tetrafluoroborate (**1**) (Fig. 1b) is computed to be an unbound state that eliminates ammonia to generate the triplet nitrenium ion to conserve spin, irrespective of whether this is the ground state of the nitrenium ion. Thus, if ISC is on par or faster than singlet photoheterolysis, a triplet state of the nitrenium ion may be formed, even though the triplet state is not the ground state. Promisingly, the parent chromophore, *p-*iodoaniline, undergoes ISC in 5 ps (see Supporting Information), suggesting that ISC for the synthesized photoprecursor **1** may be competitive with excited state bond cleavage.

In this work, we investigated the reactivity of the *p*-iodophenylnitrenium ion **2** using femtosecond transient absorption (fs-TA), nanosecond transient absorption (ns-TA) and nanosecond time-resolved Resonance Raman (ns-TR^3^) spectroscopy methods as well as computational studies. These studies resulted in direct observation of the triplet 4-iodophenylnitrenium ion ^3^**2** (*np*) giving a first look at a triplet nitrenium ion. Subsequent formation of downstream radical abstraction products provide new insight into the structure and chemical reactivity of triplet arylnitrenium ions, and more broadly provide insight into the reactivity patterns of different electronic configurations of nitrenium ions.

## Results and discussion

### Femtosecond transient absorption study of 1

**Fig. 2:**
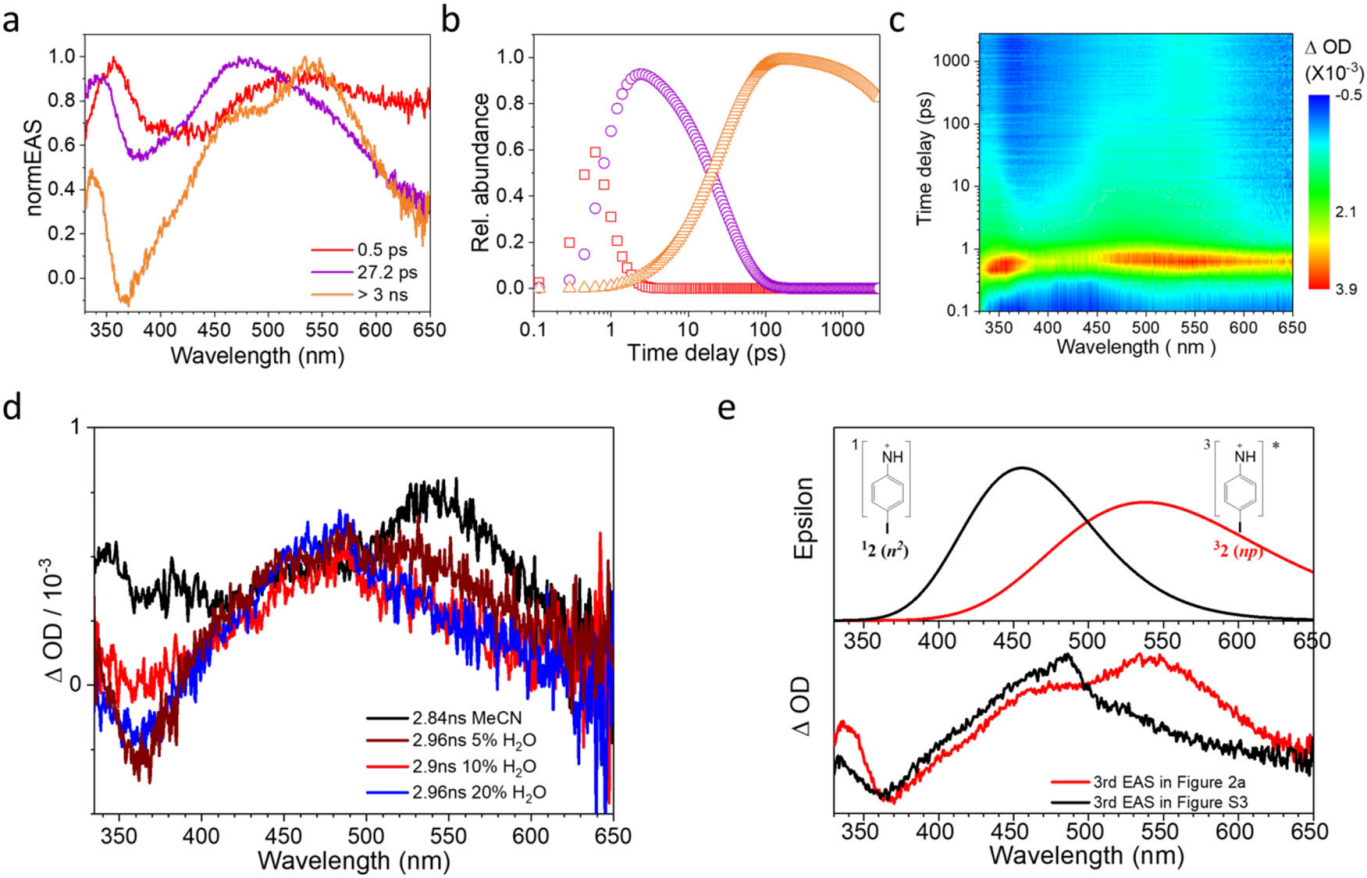
Femtosecond transient absoprtion spectroscopy (fs-TA) spectra of the precursor 1 after 267 nm excitation in MeCN. **a** normalized- Evolution Associated Difference Spectra (EAS) according to the sequential kinetic models, (**b**) time evolution of the state population obtained from the global fitting analysis and the color of the fitting trace is consistent with the EAS spectra represented in Fig. 2a, (**c**) contour plots of the time-resolved absorption spectroscopic responses, (**d**) comparison of the third EAS spectra obtained in different percentages of water in MeCN, (**e**) the comparism of the experimental data (bottom) and the calculated UV–vis spectra(top): the 3rd EAS spectrum in Fig. 2a (bottom, red) and the 3rd EAS spectrum in Supplementary Fig. 3 (bottom, black), computed UV-vis spectra of the triplet 4-iodophenylnitrenium ion ^**3**^**2** (*np*) (top, red) and closed-shell 4-iodophenylnitrenium ion ^**1**^**2** (*n*^*2*^) (top, black) obtained by CASPT2//CASSCF/PCM/cc-pVDZ calculations. The Cartesian Coordinates of the computed structures are shown in the Supplementary Data File.

**Fig. 3:**
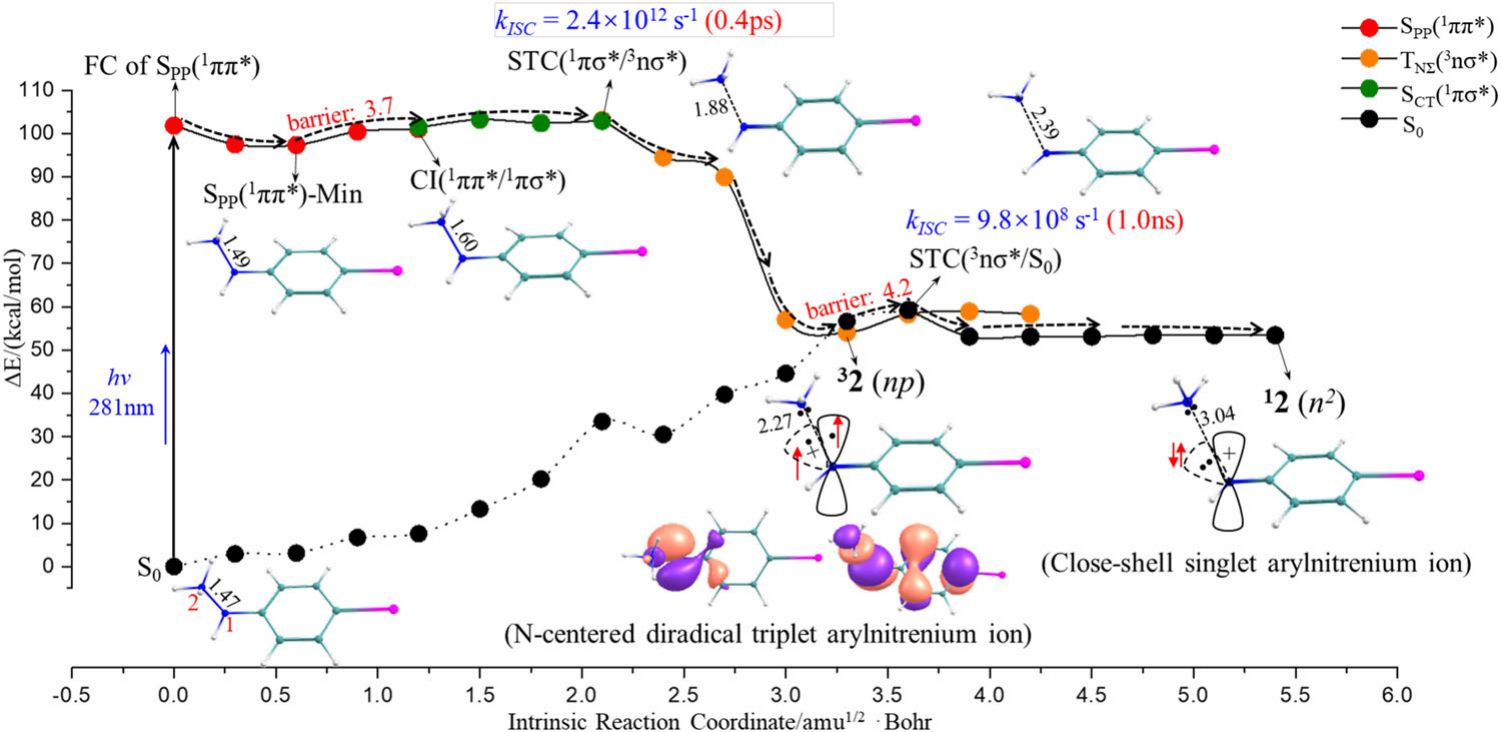
Minimum energy profiles (MEPs) for the photolysis of 1 producing the N-centered diradical ^3^2 (*np*) and closed-shell singlet ^1^2 (*n*^*2*^) along the reaction channels of the singlet and triplet states. The MEPs calculated at the CASPT2//CASSCF/PCM/cc-pVDZ level of theory, in which the line with red dots represents the S_PP_(^1^ππ*) state, the line with green dots represents the S_CT_(^1^πσ*) state, the line with orange dots represents the T_NΣ_(^3^nσ*) state and the line with black dots represents the S_0_ state. Selected stationary structures are given with their key bond (N–N) lengths in Å while the singly occupied molecular orbitals for the selective arylnitrenium ions are schematically provided. The value of 3.7 kcal/mol is the barrier required to be overcome to reach the CT(^1^ππ*/^1^πσ*) from the S_PP_(^1^ππ*)-Min, while the value of 4.2 kcal/mol is the barrier required to be overcome to arrive at the STC(^3^nσ*/S_0_) from ^**3**^**2** (*np*). The energy for each point in the MEPs and the structures for critical points are shown in the Supplementary Data File (FC Frank-Condon, STC singlet-triplet crossing; ISC intersystem crossing, CI = conical intersection).

Photolysis of **1** was examined using fs-TA experiments performed in MeCN, 5% H_2_O (MeCN), 10% H_2_O (MeCN) and 20% H_2_O (MeCN) mixed aqueous solutions after excitation by 267 nm. The 3D contour plots and global fitting results utilizing the sequential kinetics scheme^22^ from the fs-TA spectra in pure MeCN are displayed in Fig. 2a–c. As seen in Fig. 2a, b, three evolution associated difference spectra (EAS) are isolated through the global analysis with the lifetimes at 0.5 ps (red), 27 ps (purple) and > 3 ns (orange), exceeding the time-scale of our fs-TA instrument. The initial species with one absorption band at 360 nm and another broad band at 530 nm showing a lifetime at 0.5 ps can be assigned to the higher singlet excited states (S_n_) of precursor **1** and similar spectra are also observed with the presence of H_2_O (as shown in Supplementary Figs. 1–3). The internal conversion from S_n_ to S_1_ (lowest-lying singlet excited state) of precursor **1** takes place in ~0.5 ps. The singlet excited state of **1** (^**1**^**1**) features a broad visible absorption similar to that of the parent chromophore *p*-iodoaniline (See Supporting Information, Supplementary Figs. 4–6). By analogy with the first EAS species, the second EAS species (^**1**^**1**) displays hypochromatic-shifted bands at 350 nm and 470 nm with a lifetime of ~27 ps. With the decay of ^**1**^**1**, a new species appears. This species has a main absorption at 550 nm with a shoulder in MeCN that disappears upon changing the solvent from MeCN to 20% H_2_O (MeCN). As discussed later, this band is assigned to an ISC process of the ^**1**^**1** to initially form a triplet excited state of precursor ^**3**^**1**, which is an unbound state that generates the triplet nitrenium ion ^**3**^**2** (Fig. 3). According to previous studies, the neutral ammonia group was thought to be ejected easily to generate oxenium ions^23–25^ and nitrenium ions^26^ after photolysis by 267 nm. Therefore, we may anticipate a similar heterolytic scission of the N–N bond of **1** take place to give birth to the nitrenium ion ^**3**^**2**. Supplementary Fig. 3 displays the 3D contour plots and global fitting results by using the sequential kinetics scheme from the fs-TA spectra in a 20% H_2_O (MeCN) mixed aqueous solution. As expected, the first EAS and second EAS presented in Supplementary Fig. 3 look similar to the corresponded ones in Fig. 2a, which indicate the initial reaction pathway is not significantly affected by the solvent. Undoubtedly, the first EAS species could also be assigned to the S_n_ excited states of precursor **1** with a similar lifetime around 0.4 ps and the second EAS is able to assign to ^**1**^**1**.

In order to recognize the significance of the iodine presented in **1**, the photochemistry of both 2-phenylhydrazine-1-ium tetrafluoroborate (Supplementary Figs. 7, 8) and *p*-iodoaniline (Supplementary Figs. 4–6) were studied for comparison. As shown in Supplementary Fig. 7, the singlet phenyl nitrenium ion^27^ is generated after irradiation of 2-phenylhydrazine-1-ium tetrafluoroborate. However, with the presence of iodine in *p*-iodoaniline, the triplet *p*-iodoaniline is obtained with absorption bands at 400 nm and 560 nm after 14 ps (Supplementary Fig. 5) and the time constant of ISC observed for *p*-iodoaniline is 5 ps. This observation is also in good agreement with previous work reported by Hara and coworkers^28^, who pointed out that the ISC process of *p*-halogenated aniline took place in <4 ns. Thus, the presence of the iodine is anticipated to enhance the ISC process and result in generating the triplet nitrenium ion.

From the product study, the major product of **1** after irradiation in MeCN is *p*-iodoaniline. Trace amounts of *p*-phenylenediamine are also observed. In water, no photochemistry reaction is observed at all; after 1 hr irradiation, the photoprecursor is unchanged. Clearly, in MeCN the photochemistry involves breaking the N–N bond. The two possibilities for this are homolytic bond cleavage and heterolytic bond cleavage. Homolytic bond cleavage to generate the *p*-anilino radical and the ammonia radical cation can be ruled out because the radical is not observed in the transient absorption spectra. Such radicals are generally long-lived species (e.g. >nanosecond lifetime in solution) that have absorptions mostly in the UV region of the optical spectrum (computational predicted UV-vis absorption spectra can be found in Supplementary Fig. 9 while the spectrum for related anilino radicals is known^29^). The other possibility, heterolytic bond cleavage, could occur either in the singlet excited state or the triplet excited state, which would generate the ground-state singlet nitrenium ion or the excited triplet nitrenium ion, respectively, to conserve spin.

The band associated with S_1_ evolves over 27 ps into two absorptions that both live longer than the time limitation of the femtosecond instrument (3 ns). One of the absorptions is centered ~550 nm while the less intense absorption is centered ~470 nm. Interestingly, as the water content of the solution is increased from pure MeCN to 20% H_2_O (MeCN), the 550 nm absorption disappears while the peak at 470 nm grows to take its place (Fig. 2c). These results are consistent with competitive formation of two intermediates from S_1_ in MeCN (with the longer-wavelength absorbing transient converting over longer timescales to the shorter-wavelength absorbing transient), but in water the channel creating the 550 nm peak becomes disfavored while the channel creating the 470 nm peak takes its place. This could be due to the hydrogen bonding between the water and the lone pair on the nitrogen, which inhibits the ISC process.

So what is the identity of the carrier of the peak at 550 nm, which is not formed in water? The precursor to this band is the singlet excited state while this 550 nm intermediate subsequently decays over 1.1 μs into the peak at 470 nm. The transient carrier of the 470 nm absorption then decays over 15 μs into the *p*-iodoaniline radical cation (seen also in the ns-TR^3^ experiments, described later).

**Fig. 4:**
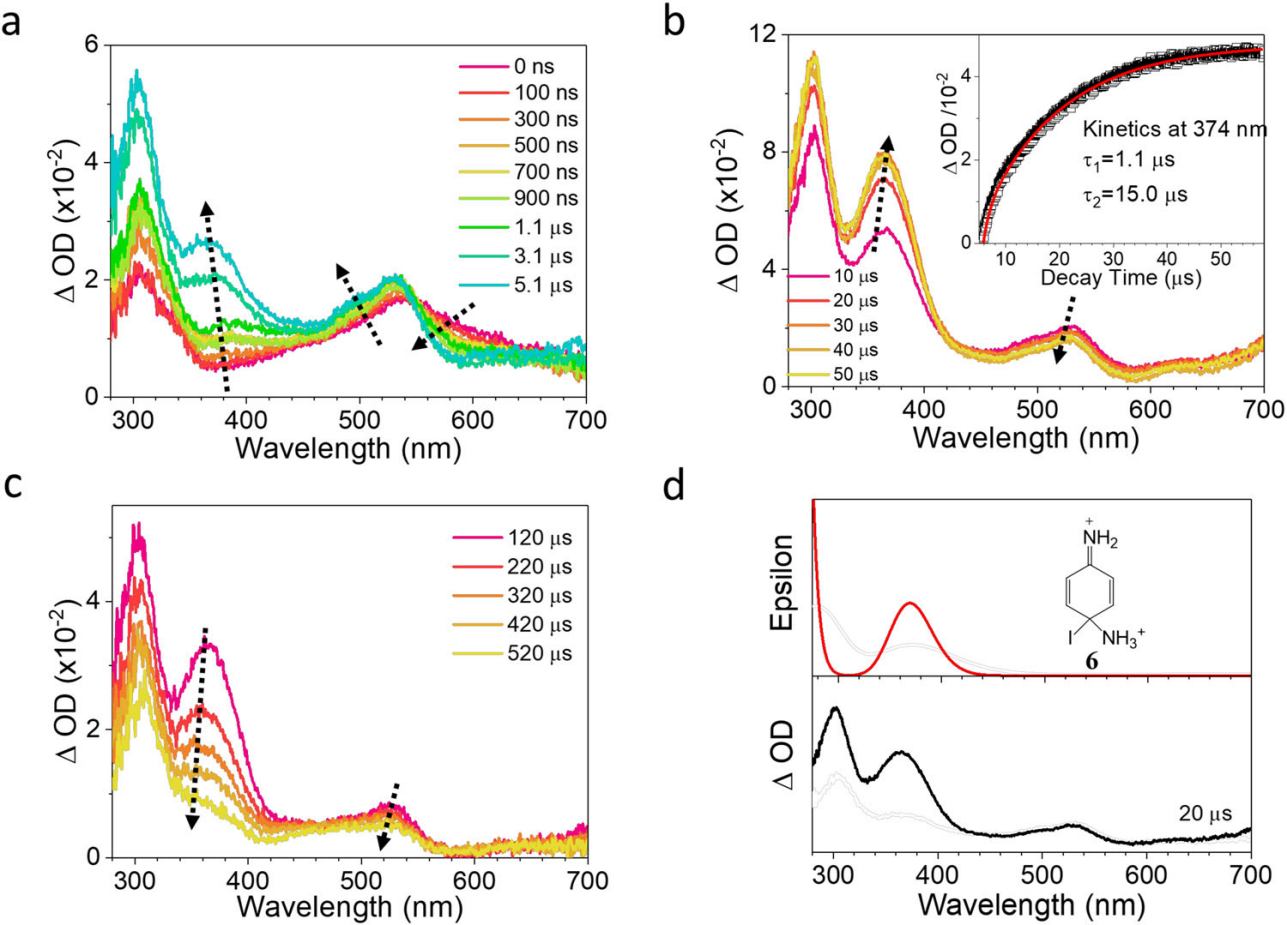
Nanosecond transient absorption spectroscopy (ns-TA) spectra of the precursor 1 after 266 nm excitation in MeCN. **a**–**c** spectra obtained at different delay times, and the kinetics at 374 nm (gray square) with fitting to the experimental data points (red solid line) was inserted in (**b**), (**d**) comparison between the TA spectrum at 20 μs (bottom, black) and the predicted UV–vis spectrum of dication **6** (top, red and with the structure shown) using TDDFT/M06-2X calculation. The Cartesian coordinates of the computed structures are shown in the Supplementary Data File.

Several pieces of evidence point to the assignment of the 550 nm band to the triplet nitrenium ion ^3^**2** and the 470 nm band as the singlet nitrenium ion ^1^**2**. Thus, the full photochemical pathway in MeCN involves the S_1_ state of **1** partitioning between ISC to generate the triplet nitrenium ion ^3^**2** (550 nm) and heterolysis to generate the singlet nitrenium ion ^1^**2** (470 nm). Subsequently, the excited triplet nitrenium ion undergoes ISC to convert into the ground singlet state of the nitrenium ion, which results in the increasing intensity of the band at 470 nm till about 5 μs time delay (Fig. 4a). In water, ISC is inhibited and S_1_ exclusively forms the singlet nitrenium ion. Similar effects of solvent on the rate of ISC have been noted previously^30^. Note that the triplet excited state of **1** is computed to be an unbound state by CASPT2 computations (Fig. 3), converting without a barrier to the triplet nitrenium ion ^3^**2** and ammonia, suggesting the triplet excited state of the photoprecursor is either not an intermediate or so short-lived that it would not be directly observed. An ISC event occurring in tens of picoseconds is normal for a spin-orbit coupling (SOC) allowed ^1^πσ*→^3^nσ* conversion, similar with the SOC-allowed ISC rate for the ^1^nπ*→^3^ππ* intersystem crossing of benzophenone^31,32^. However, the parent chromophore *p*-iodoaniline undergoes ISC in 5 ps (Supplementary Fig. 5) in MeCN, suggesting that ISC for this chromophore is accelerated by the heavy iodine atom. An ISC for **1** of ~27 ps is consistent with that time-scale.

These assignments of the 550 nm to the triplet excited nitrenium ion ^3^**2** and the 470 nm band to the singlet nitrenium ion ^1^**2** is supported by the CASPT2/cc-pVDZ predicted UV–vis bands of the triplet and singlet nitrenium ions (Fig. 2e, Supplementary Figs. 10, 11 and Supplementary Tables 1, 2). Interestingly, the absorption of the triplet nitrenium ion is almost identical to that of the *p*-iodoaniline radical cation observed at much later time delays in the ns-TA. It would be expected that the *p*-iodoaniline radical cation and the triplet nitrenium ion would have similar absorption profiles, since the π orbital occupations are identical. Thus, the fact that the 550 nm transient has a similar absorption to the *p*-iodoaniline radical cation supports the assignment to an open-shell nitrenium ion. This represents the first direct observation of a triplet arylnitrenium ion by time-resolved spectroscopy.

### CASPT2 Computational Analysis of the Photochemical Mechanisms of 1

To investigate the photogeneration of the arylnitrenium ions, the optical properties of **1** were computationally analyzed. As shown in Supplementary Table 3, a bright spectroscopic state is determined computationally to originate from charge-transfer (CT) excitation along the desired direction from the π orbital of *p*-iodophenyl moiety to the N–N σ* orbital. However, the vertical excitation of S_0_→ S_CT_(^1^πσ*) transition energy for **1** is more than 140.0 kcal/mol and goes beyond the range of the photoexcitation wavelength (267 nm) in this work. As shown in Fig. 3, a slight elongation of the N–N bond in S_PP_(^1^ππ*) state which originates from an electron promotion from the *p*-iodophenyl centered π orbital to its π* orbital causes a drastic decrease in the energy level of the bright S_CT_(^1^πσ*) state, leading to a conical intersection (CI) between these two states, referred to as CI (^1^ππ*/^1^πσ*). The existence of CI(^1^ππ*/^1^πσ*) provides an effective non-adiabatic relay to allow an instantaneous CT from the phenyl moiety to the NH_3_^+^ moiety.

Once one electron populates in the repulsive σ* orbital, the N–N bond fission is triggered instantaneously along a flat pathway in the S_CT_(^1^πσ*) state. In this relaxation process, the NH_3_^+^ gradually escapes from the bondage of the *p*-iodophenyl moiety, thereby generating the isolated ‧NH_3_^+^ with a radical cation configuration. Mulliken population analyses demonstrate that the ‧NH_3_^+^ is gradually being neutralized by the charge redistribution of the ejected electron from the *p*-iodophenyl group in the S_CT_ (^1^πσ*) state. These changes can be evidenced by the positive charge evolutions of the NH_3_ fragment along the relaxation pathway, i.e., S_0_-Min (+0.84)→CI(^1^ππ*/^1^πσ*) (+0.67)→STC(^1^πσ*/^3^nσ*) (+0.48)→^**3**^**2** (*np*) (+0.11) (Supplementary Fig. 12). Based on the above structural deformation and charge redistribution, the triplet state channel switches on through a fast ISC, which can be characterized computationally by a singlet-triplet crossing (STC) between the ^1^πσ* and ^3^nσ* states, i.e., STC(^1^πσ*/^3^nσ*) at 1.88 Å N–N distance. The presence of the iodine heavy atom significantly improves the SOC and thus accelerates the ISC rate to the picosecond time-scale (the calculated rate: *k*_ISC_ = 2.4 × 10^12^ s^−1^), facilitating the generation of nitrenium ion in the triplet state.

The departure of ‧NH_3_^+^ closely cooperates with the effective intra/inter-molecular charge transfer in a concerted manner along the relaxation path of ^3^nσ* state. As a result, one unpaired electron distributes in the N moiety of arylnitrenium ion along the vertical direction of the molecular plane. This can certainly be ascribed to the photoinduced N–N bond fission with concomitant of the electron pairing around ‧NH_3_^+^ moiety, thus generating the neutral:NH_3_. The character of the singly occupied orbital evolves into the N-centered *p* orbital from the initial σ nature in the ^3^nσ* precursor state due to the departure of the ammonia moiety. Meanwhile, another unpaired electron is left in the N moiety of the arylnitrenium ion along the parallel direction of the molecular plane, which initially originates from the lone pair of the nitrogen. Accordingly, the open-shell species is eventually generated to show a N-centered diradical configuration that has been denoted as the ^**3**^**2** (*np*) arylnitrenium ion in the discussions above. As shown in Fig. 3, the generation of ^**3**^**2** (*np*) can be characterized by a rapidly falling relaxation pathway of the triplet ^3^nσ* state with more than 47.0 kcal/mol energy decrease, which is qualitatively correlated with the dynamically observed experimental results as discussed above. All the computational pieces of evidence unambiguously reveal that the N-centered diradical ^**3**^**2** is the predominant triplet state product, whose generation can be controlled dynamically and thermodynamically by the enhanced SOC and the electron-donating abilities in the presence of the relativistic effects of the iodine.

The possible generation of the closed-shell species was also examined and this search is shown in Fig. 3. With the formation of the ^**3**^**2** (*np*) arylnitrenium ion, the energy level of the S_0_ state gradually ascends and eventually matches with the ^3^nσ* state, leading to another STC(^3^nσ*/S_0_) at a 2.39 Å N–N distance. The determined non-adiabatic relay may allow a spin-forbidden electron pairing in the N-centered n orbital, producing the closed-shell arylnitrenium ion ^**1**^**2** (*n*^*2*^). However, the efficiency for the formation of ^**1**^**2** (*n*^*2*^) is quite low, since the ISC rate for ^3^nσ*→S_0_ transition has been estimated to be 9.8 × 10^8^ s^−1^. The calculated kinetics data closely correlates with the experimental observations that the closed-shell species can be generated within 1.1 μs. Moreover, a small barrier (4.2 kcal/mol) has to be overcome to produce ^**1**^**2** (*n*^*2*^). All of the computational pieces of evidence demonstrate that closed-shell species ^**1**^**2** (*n*^*2*^) is minor and unlikely competes with the generation of ^**3**^**2** (*np*) along the triplet state pathway.

Careful analyses based on the structures of the S_CT_(^1^πσ*) state demonstrate that the S_NΣ_(^1^nσ*) state is also energetically degenerate with the S_CT_(^1^πσ*) state at a 1.78 Å N–N distance, denoted as CI(^1^πσ*/^1^nσ*). Consequently, multiple reaction channels are possible and the singlet form of **2** can be generated along the pathway of the singlet state. Indeed, the initial decay in the S_NΣ_(^1^nσ*) state proceeds smoothly along a downhill pathway after the occurrence of the fast interconversion (^1^πσ*→^1^nσ*) via an effective relay of CI(^1^πσ*/^1^nσ*). However, another conical intersection between the ^1^nσ* and ground states, denoted as CI(^1^nσ*/S_0_), is encountered at a 2.47 Å N–N distance before the system relaxes to the minimum of the S_NΣ_(^1^nσ*) state, S_NΣ_(^1^nσ*)-Min. As shown in Supplementary Fig. 13, two de-excitation pathways were determined through the non-adiabatic relay of CI(^1^nσ*/S_0_). On the one hand, the non-productive decay takes place with high possibility through the radical recombination associated with the rebirth of the N–N bond along a downhill pathway in the S_0_ state. As a result, the photo-initiated precursor recovers its original state of **1**, which undergoes further photoexcitation and thus reduces greatly the quantum yield of the singlet state. On the other hand, the radical pairing occurs in the N-centered n orbital with the departure of the ammonia moiety, producing the closed-shell singlet arylnitrenium ion of ^**1**^**2** (*n*^*2*^). Considering the presence of the loose channel for the quantum yield of the singlet state due to the spin-allowed radical pairing, the generation of the excited triplet species ^**3**^**2** is predominant over the concomitant singlet decay channel.

Thus, these computations give mechanistic plausibility to the formation of the triplet nitrenium ion. The S_1_ geometry of the photoprecursor **1** features an elongated N–N bond of 1.49 Å, which is 0.02 Å longer than the bond length in the ground state. Continuing further along the N–N stretch coordinate the excited state changes character from a ππ* state to a πσ* state. Further along the N–N stretch coordinate still is a STC. An ISC event here, promoted by the heavy atom effects, generates an unbound triplet excited state of the photoprecursor that spontaneously dissociates to the triplet nπ phenylnitrenium ion and ammonia. Note that the transition state for reaching this STC is lower in energy than the Frank-Condon S_1_ energy, so it is plausible this STC could be reached on an ultrafast time-scale. However, any STC of the photoprecursor would be expected to spontaneously form the triplet nitrenium ion as well, as optimizing the triplet state of **1** starting from the geometry of the singlet photoprecursor leads to spontaneous dissociation to the triplet nitrenium ion and ammonia.

### Nanosecond time-resolved spectroscopic study

In order to provide the integrated spectrum of the later species after 3 ns, ns-TA was employed to study the reaction pathways of precursor **1** after irradiation (Fig. 4). As discussed above, the full photochemical pathway in MeCN involves the S_1_ state of **1** partitioning between ISC to generate the triplet nitrenium ion ^**3**^**2** (550 nm) and heterolysis to generate the singlet nitrenium ion ^**1**^**2** (470 nm). Subsequently, the excited triplet nitrenium ion undergoes ISC to convert into the ground singlet state of the nitrenium ion, which results in the increasing intensity of the 470 nm bandtill 5 μs time delay (Fig. 4a). The kinetics at 374 nm is able to be fitted by a biexponential function with two time constants (1.1 µs and 15.0 µs) as inserted in Fig. 4b. Thus, the first process is mainly due to the ISC process from the triplet nitrenium ion ^**3**^**2** to the singlet nitrenium ion ^**1**^**2**. The second process could be assigned to the generation of *p*-iodoaniline radical cation **4**, which has support from Falvey and coworkers^26^, who showed that ns-TA of protonated diarylhydrazines led to the singlet diarylnitrenium ions, which decayed into the *p*-iodoaniline radical cation **4**. The authors attributed this transformation to a proton and electron transfer from unreacted starting material.

In addition, the absorption intensity at 540 nm changes little, which indicates the proton and electron transfer processes could take place more slowly (15 µs). Other than the proton and electron transfer processes, the leaving group (:NH_3_) can attacke the singlet nitrenium ion ^**1**^**2** (resonance structure) to form the diaminium species (refer to as **6**) adopting the cyclohexa-2,5-diene structure, which is also a dication intermediate. Then, as proposed by Tee and coworkers previously^33,34^, the dehalogenation and deprotonation reactions occur for species **6** to form the *p*-phenylenediamine (referred to as **7**). Furthermore, the observation of *p*-phenylenediamine in the product analysis in Supplementary Fig. 14 also supports this hypothesis. Time-dependent Density Functional Theory (TDDFT) calculations were used to predict the UV–vis absoprtion spectrum of the dication **6**, and showed good agreement with the TA spectrum obtained at 20 µs (Fig. 4d). Therefore, one of the second processes observed in Fig. 4a with a lifetime around 15 µs can be assigned to the formation of dication **6**, which ultimately forms the *p*-phenylenediamine product **7**.

**Fig. 5:**
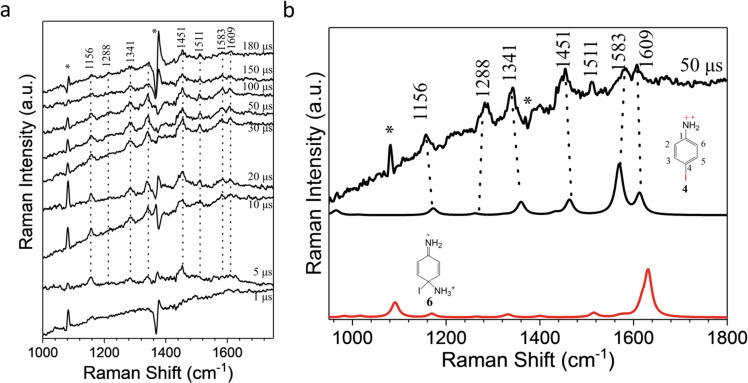
Nanosecond time-resolved resonance Raman spectroscopy (ns-TR^3^) spectra of the precursor 1 in pure MeCN by employing a 355 nm probe laser and a 240 nm pump laser. **a** spectra obtained at different delay times. Asterisks represent solvent substraction artifacts, (**b**) spectrum at 50 µs (top), computed normal Raman spectra of the radical cation **4** (middle, with the structure shown) and dication **6** (bottom, with the structure shown) using DFT/M06-2X calcaultion. Asterisks represent solvent substraction artifacts. The Cartesian Coordinates of the computed structures are shown in the Supplementary Data File.

In order to discover the vibrational spectroscopic character of the species involved in the ns-TA experiments, we employed ns-TR^3^ measurements by using 355 nm as the probe wavelength in pure MeCN in Fig. 5a. As shown in Fig. 5a, only one species is obtained and it increases from 1 µs to 50 µs and decreases slowly afterward and this is consistent with the evolution of the longer lived species whose generation lifetime is 15 µs observed in Fig. 4b. Unfortunately, the finger print information of triplet nitrenium ion ^**3**^**2** was not observed throughout this experiment. As discussed above, the radical cation **4** and dication **6** could be formed slowly, therefore, the DFT calculation here is used to predict the Raman spectra for the radical cation species **4** and dication **6**. The comparison of the spectra between the experimental results and computational results is displayed in Fig. 5b. Resonance enhancement of the TR^3^ spectra can result in differences in the relative intensities of the Raman spectra between the experimental and the calculated results, particularly when the chromophore for the enhancement is somewhat delocalized. Figure 5b indicates both the Raman vibrational frequencies and the relative intensities computed for the radical cation intermedate **4** is consitent with the TR^3^ spectrum. Therefore, we can assign the TR^3^ spectrum to the radical cation species **4**. In Fig. 5b, six major Raman shifts at 1609, 1583, 1451, 1341, 1288 and 1156 cm^−1^ are observed. According to the vibrational modes simulated by the DFT calculation, we are able to assign the experimental Raman bands accordingly. The 1609 and 1451 cm^−1^ Raman bands are mainly due to the stretching mode of the C1-N bond. As displayed in Supplementary Fig. 15, the bond length of the C1-N bond is predicted to be 1.34 Å due to the delocalization of the radical on the phenyl ring and shows intense vibrational properities as a double bond. The bands at 1609 cm^−1^ also has some contributions from the sicissoring modes of the H-N-H bonds. The 1583 cm^−1^ Raman feature is associated with the enhanced stretching mode of the C2–C3 and C5–C6 bonds and some in-plane rocking vibrational modes of the C–H bonds on the phenyl ring. The 1341 and 1288 cm^−1^ Raman bands can be assigned to the C–H and N–H rocking modes. The 1156 cm^−1^ Raman band is due to the scissoring modes of the C–H bands on the phenyl ring.

### Photoproduct studies of 1

Photoproduct studies of 1 were carried out in deuterated MeCN to obtain the time-resolved photolysis progress by reading ^1^H NMR (Supplementary Fig. 14). The LCMS data collects the photolysis information of **1** using the corresponding non-deuterated solvent (Supplementary Fig. 16). It clearly demonstrates the appearance of the reduced *p*-iodoaniline **5** (protonated) along with singlet adducts (**7** and ortho-nucleophilic adduct, observed in ^1^H NMR but not detected by LCMS), indicating the generation of the excited triplet species ^**3**^**2** competing against a concomitant singlet decay channel. However, this photoprecusor appears to have deficient photo activity in water (Supplementary Fig. 17) as it exhibits no noticeable reaction over 70 min of irradiation in water, suggesting the photolysis of this precursor is highly solvent-dependent. The photoproducts show agreement with the proposed photochemical reaction pathways and further confirm that this *n,p* triplet excited nitrenium ion exhibits diradical reactivity.

**Fig. 6:**
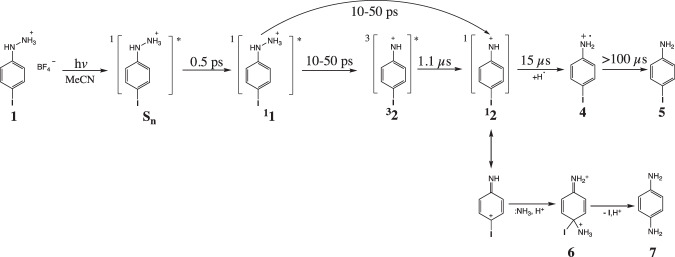
The proposed photoinduced reaction mechanisms after 267 nm photoexcitation of 1 in MeCN. After the photoexcitation, the ^**1**^**1** partitions between heterolysis to generate the singlet nitrenium ion ^**1**^**2** and intersystem crossing (ISC) as a result of the heavy iodine atom followed by a spontaneous heterolysis process to generate the triplet nitrenium ion ^**3**^**2**. The ^**3**^**2** undergoes ISC with in 15 μs to generate the ^**1**^**2**, which ultimately undergoes proton and electron transfer to generate a long-lived radical cation 4 that further reacts on the microsecond time scale to generate the reduced p-iodoaniline 5 as the major photoproduct.

In conclusion, the novel precursor for the production of the triplet *p*-iodophenylnitrenium ion ^3^**2** (*np*), 2-(4-iodo-phenyl)hydrazin-1-ium tetrafluoroborate **1**, was investigated by using time-resolved spectroscopic experiments (fs-TA, ns-TA and ns-TR^3^) as well as the theoretical calculations (DFT, TDDFT and CASPT2//CASSCF). These results allow us to provide reaction pathways for **1** after irradiation in MeCN as shown in Fig. 6. After excitation of precursor **1**, both the triplet *p*-iodophenylnitrenium ion ^3^**2** and singlet *p*-iodophenylnitrenium ion ^1^**2** were produced after 10-50 ps, and their reactivity were influenced by their electronic configurations. Then, the triplet arylnitrenium ion ^3^**2** decayed via ISC into the closed-shell singlet ground state ion ^1^**2**, which could undergo proton and electron transfer processes to form the radical cation species **4** and generate the p-iodoaniline 5 as the major photoproduct. Alternatively, the singlet arylnitrenium ion ^1^**2** generate the *p*-phenylenediamine **7** through the dication species **6**. Therefore, controlling the electronic configurations of arylnitrenium ions can have potential to provide useful new synthetic transformations. Apparently, the structure and properties of the arylnitrenium ions are able to influence their attack position towards guanosine derivatives according to our previous studies^35,36^, further studies will be employed on studying the photoinduced reactions between the precursor **1** and selected nucleobases.

## Methods

### Materials

Detailed synthetic procedures and characterization data of precursor **1** are given in Supplementary Figs. 16, 18–20. Spectroscopic grade MeCN was utilized for the preparation of the sample solutions. All of the mixed solvent ratios are of volume ratios unless indicated otherwise.

### Fs-TA and ns-TA experiments

The experimental setups and method for those experiments have been described in our previous studies^30^.

The fs-TA experiments were conducted on a commercialized Helios pump-probe system (Ultrafast System) with the femtosecond laser beam from the regenerative amplified Ti:sapphire laser system (Spectra Physics, Spitfire Pro). The laser light (120 fs, 800 nm) was then split into two beams with one using as the pump beam and one as the probe beam. For the present experiments, the wavelength of the pump beam was set as 267 nm (the third harmonic of the fundamental 800 nm), while the probe beam passed through a CaF_2_ crystal and generated a white-light continuum (330–650 nm). After the solution was photoexcited by the pump light, the time-delayed probe beam (controlled by the optical delay rail with a maximum temporal delay at 3.3 ns) would be passing through the photoexcited solution and the TA signals were then collected by the detector. A reference probe beam was also used to obtain better signal-to-noise ratio. The 80 mL sample solutions were prepared with absorbance around 1 at 267 nm and circulated in a 2 mm path-length quartz cuvette.

The ns-TA experiments were performed on a LP920 laser flash spectrometer by Edinburgh Instruments Ltd. Briefly, the continuous probe light ranged from 280 to 800 nm was produced by a 450 W ozone free Xe arc lamp, and the 266 nm pump beam was obtained from the fourth harmonic output of an Nd:YAG laser. The 80 mL sample solutions with an absorption of ∼1 at 266 nm were prepared in a flowing 1 cm path-length quartz cuvette. The transmitted signals were collected by a photomultiplier detector (for kinetics mode) and an array detector (for spectral mode).

### Ns-TR^3^ measurements

The ns-TR^3^ measurements were also described in detail previously^30,37^. The ns-TR^3^ was a hand-made technique using electronic time-delay stage to control the pump-probe laser beam. The wavelengths of the pump and probe laser beam were selected as 240 nm (first anti-Stokes hydrogen Raman-shifted laser line from the fourth harmonic of a Nd:YAG nanosecond pulsed laser) and 355 nm (third harmonic of a Nd:YAG nanosecond pulsed laser) for use in the current presented experiments. These two laser beams co-focused spatially in the same spot with the size of the pump laser slightly bigger than that of the probe laser beam. After photoexcitation, the Raman scattered signals of the transient species were collected by a liquid-nitrogen-cooled charge-coupled device (CCD) detector using backscattering geometry. The Raman bands of ACN were used to calibrate the Raman shifts with an estimated uncertainty of 5 cm^−1^. The 50 mL solutions with absorbance of ∼1 at 266 nm in a 2 mm path-length cuvette were prepared for use. The TR^3^ spectra were obtained by the subtraction of a resonance Raman spectrum with a negative time delay of −100 ns (probe-before-pump spectrum) from the resonance Raman spectra with positive time delays (pump − probe spectrum).

### DFT and time-dependent DFT (denoted as TDDFT) computations

The DFT computations employed the M06-2X as the methods, theoretical calculations on iodine-containing molecules, is obtained from the basis set exchange database^38–40^. A simulated band width for the spectra was determined using a Lorentzian of 10 cm^−1^ band width for the vibrational band frequencies. A 0.97 scaling factor was used to scale the vibrational frequencies. The electronic absorption spectra of the compounds were simulated by TDDFT method^27,41^, by solving the lowest 50 allowed vertical electronic transitions from the ground state. To simulate solvent broadening, each of the electronic transitions was represented by a Gaussian band shape with a half-width of 2500 cm^−1^. More details can be found in our previous studies^30^. The Gaussian 09 program suite^42^ was employed and more details were discussed previously^30^.

### CASPT2//CASSCF calculations

In the past, the hybrid CASPT2//CASSCF method^43–46^ has been verified to describe accurately the properties of ground and excited states of molecules, including also those excited states with charge-transfer (CT) character and strong spin-orbit coupling effects, as well as their electronic spectra^47–51^. To interpret the mechanism for the generation of the triplet arylnitrenium ion ^3^**2** (*np*) and the closed-shell singlet arylnitrenium ion ^**1**^**2** (*n*^*2*^), the ab initio calculations were primarily performed at the CASSCF level of theory with a total of 10 electrons in 8 active orbitals (10e/8o). The two low-lying π and corresponding π* orbitals, the related n orbitals and the σ/σ* orbitals are contained in the active space (Supplementary Fig. 21). Meanwhile, the energy-consistent relativistic pseudopotential basis sets (ECP46MWB) were applied for iodine atom to account for the most important relativistic effects and cc-pVDZ basis sets were applied for other atoms.

All of the critical points in their singlet excited states were obtained by full system state-averaged CASSCF optimizations using a two-root equally weighted (0.5:0.5) approach, whereas the single-root optimizations were adopted for the triplet excited states and the ground states. The minimum energy profiles (MEPs) were mapped by intrinsic reaction coordinate (IRC) computations to connect the critical points in several possible excited and ground states^52,53^. Considering the effects of dynamic electron correlation, the single-point energy of the optimized geometries was recalculated utilizing the second-order perturbation method (CASPT2) with a five-root state-averaged CASSCF zeroth-order wave function.

When STCs are optimized by using the SA-CASSCF procedure, the energy of the highest of the two states considered is gradually minimized by IRC calculations along the mass-weighted cartesian coordinates (rather than the predefined ones). The geometries of STC(^1^πσ*/^3^nσ*) and STC(^3^nσ*/S_0_) were obtained by 2-root-SA(^1^πσ* and S_0_)-CASSCF and single root triplet IRC optimizations, respectively. Whereas, the corresponding singlet and triplet energies based on the optimized STC structures were separately recalculated at the 5-root-SA-CASSCF/CAPT2 levels to consider the involved electron correlation. These refined energies were used to examine the energetic gaps between the target state and the projected one. The STC structures are finally defined by the smallest energy difference along the IRC relaxation paths. The adopted scheme for searching for CIs is very similar to the case of the STC optimizations. In principle, the geometries and the corresponding energies were treated separately. The CI geometries were obtained by IRC optimizations (2-root-SA-CASSCF) and the position of the CIs was determined by using the smallest energy gap between the target state and the projected one (CASPT2 with 5 states). It is noteworthy to point out that the obtained STCs or CIs tend to the points of the smallest energy gaps along the IRC pathway instead of the global minimum CI of the whole potential surface. The vertical excitation energies and the corresponding oscillator strengths (*f*) for different transitions of the precursor **1**^3^,**2** (*np*) and ^1^**2** (*n*^*2*^) were respectively obtained from the state-averaged CASSCF state interaction (CASSI)^54^ at their minimum (For details see Tables S1–S3 and Supplementary Figs. 17, 18). In addition, the solvent effect was included using the polarizable continuum model (PCM) for the acetonitrile matrix for all optimizations, IRC and CASPT2 computations. All of these preceding computations were done making use of Gaussian^42^ and Molcas program packages^54^.

### Product studies

The sample in a quartz NMR tube was dissolved in a deuterated solvent and irradiated with 254 nm UV light from a mercury vapor lamp for different desired time durations in a Rayonet photoreactor. Spectra were taken with consistent ^1^H NMR characterization parameters. Comparison between the ^1^H NMR spectra of the sample and the standard chemicals also clarified the peak assignments and the products generated. For LC-MS experiments, the sample was dissolved in the corresponding non-deuterated solvent and irradiated for the same length of time for further product confirmation.

## Supplementary information

Supplementary Information

Description of Additional Supplementary Files

Supplementary Data 1

Supplementary Data 2

## Data Availability

All the data supporting the findings of this study are available within the article and the supplementary information files. Absolute energies are found in Supplementary Data 1 while Cartesian coordinates are found in Supplementary Data 2.
