# Supplementary material for: Generation and direct observation of a triplet arylnitrenium ion
Source: Nat Commun. 2022 Jun 16;13:3458. doi: 10.1038/s41467-022-31091-z (PMC9203820; doi:10.1038/s41467-022-31091-z)
Supplement: Supplementary file 1 — Supplementary Information [file 41467_2022_31091_MOESM1_ESM.pdf]

Supplementary Information

Generation and Direct Observation of a Triplet Arylnitrenium Ion

Lili Du et al

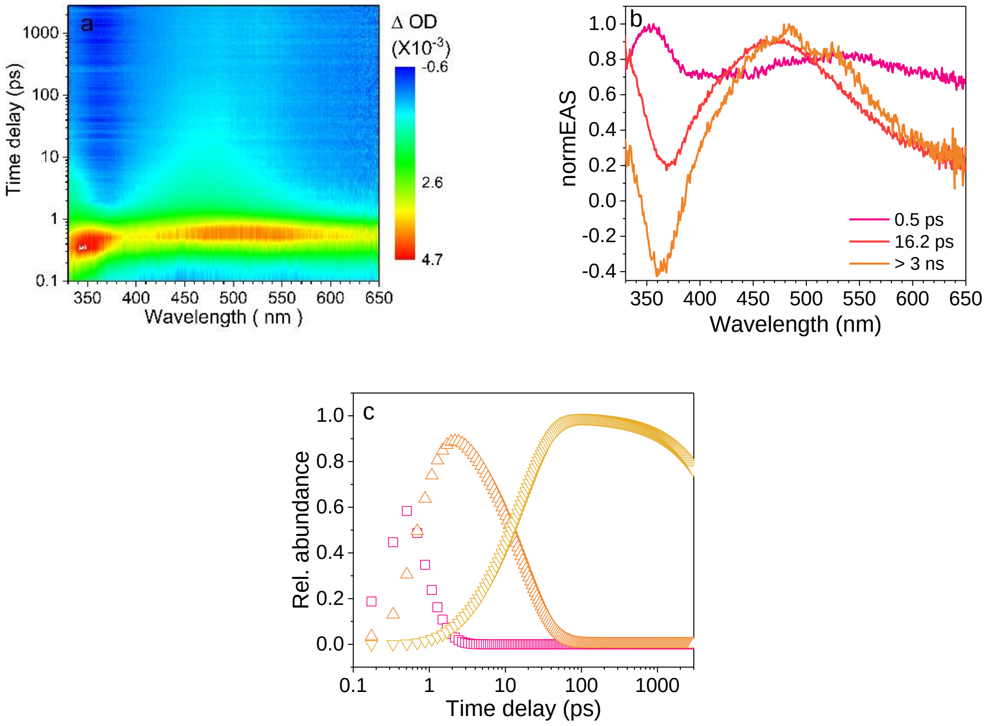

Supplementary Figure 1. **Femtosecond time-resolved transient absorption spectroscopy (fs-TA) of the precursor 1 after 267 nm excitation in 5% H_2O (MeCN).** (a), contour plots of the time-resolved absorption spectroscopic responses, (b) normalized evolution-associated difference spectra (EAS) according to the sequential kinetic models, (c) time evolution of the state populations obtained from the global fitting analysis.

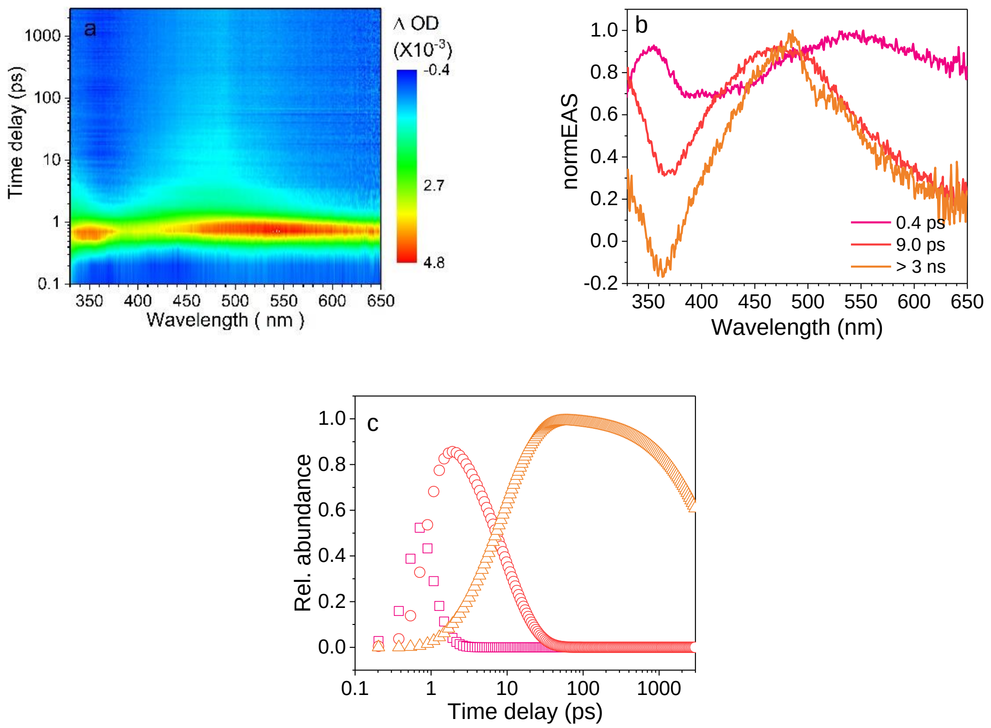

Supplementary Figure 2. **Fs-TA of the precursor 1 after 267 nm excitation in 10% H_2O (MeCN)** (a), contour plots of the time-resolved absorption spectroscopic responses, (b) normalized-EAS according to the sequential kinetic models, (c) time evolution of the state populations obtained from the global fitting analysis.

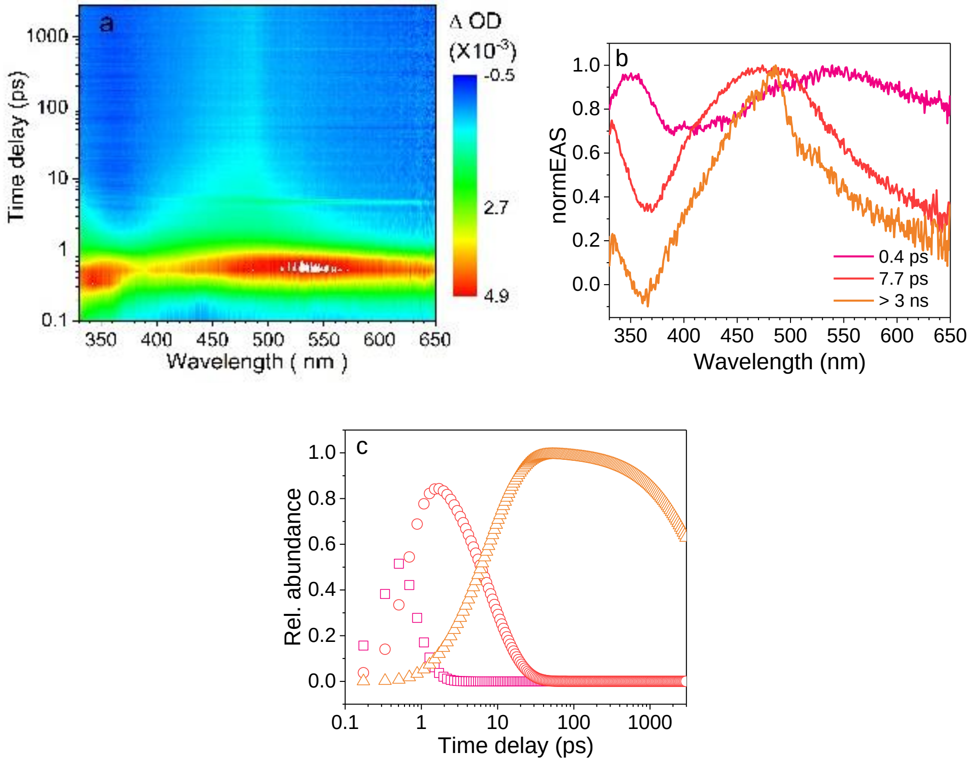

Supplementary Figure 3. Fs-TA of the precursor 1 after 267 nm excitation in 20% H_2O (MeCN) (a), contour plots of the time-resolved absorption spectroscopic responses, (b) normalized-EAS according to the sequential kinetic models, (c) time evolution of state population obtained from the global fitting analysis.

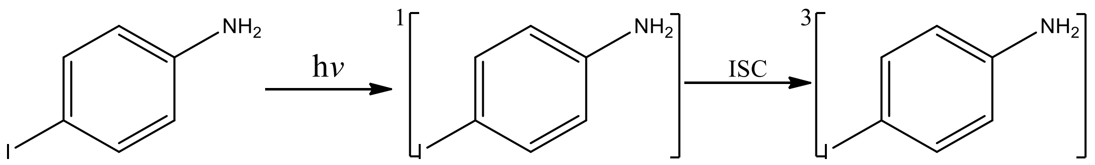

Supplementary Figure 4. **Proposed photochemical processes of *p*-iodoaniline.** After excitation, the singlet excited state of *p*-iodoaniline goes through the intersystem crossing (ISC) process to produce the triplet *p*-iodoaniline due to the heavy atom effect.

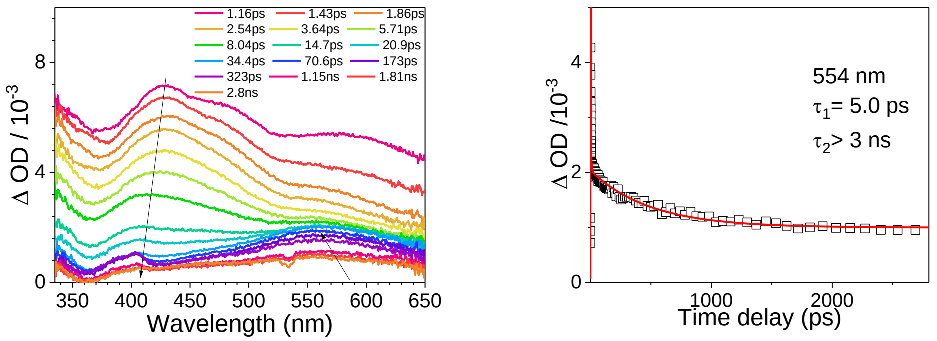

Supplementary Figure 5. Fs-TA of the *p*-iodoaniline after 267 nm excitation in MeCN (left), kinetics at 554 nm with fitting results (right).

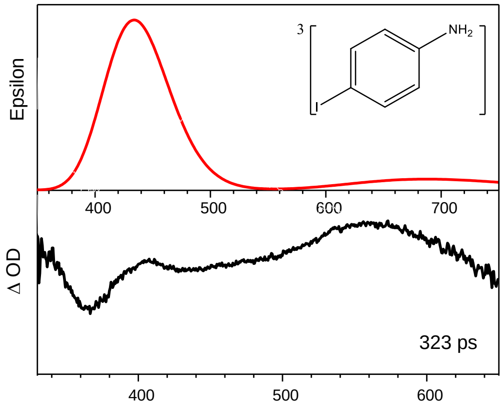

Supplementary Figure 6. The fs-TA spectrum at 323 ps of Supplementary Figure 5 (bottom, black) and computed UV-vis spectra of the triplet 4-iodoaniline (top, red).

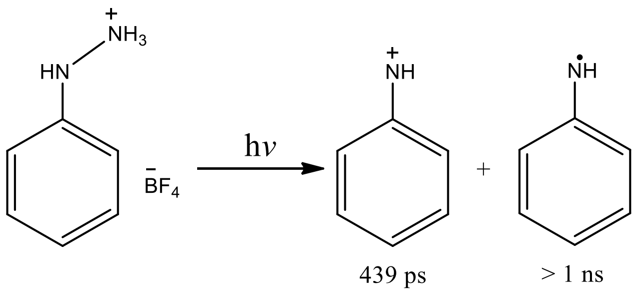

Supplementary Figure 7. Proposed photochemical processes of 2-phenylhydrazine-1-ium tetrafluoroborate. After excitation, the singlet phenyl nitrenium ion is generated with a lifetime around 439 ps and phenyl radical is also observed with a lifetime > 1 ns.

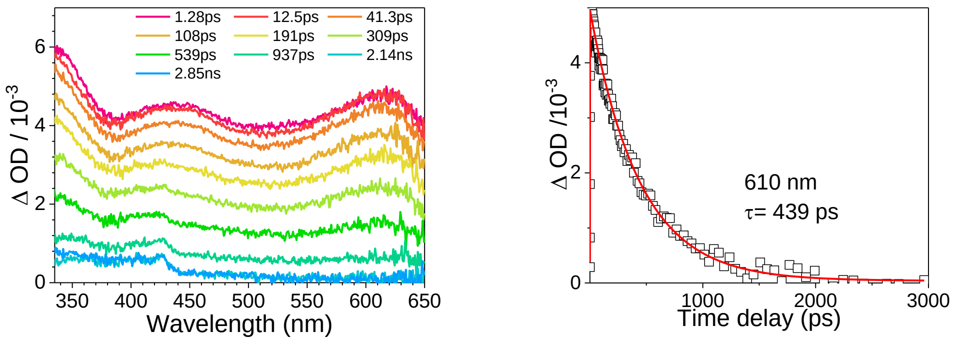

Supplementary Figure 8. Fs-TA of the 2-phenylhydrazine-1-ium tetrafluoroborate after 267 nm excitation in MeCN (left), kinetics at 610 nm with fitting results (right).

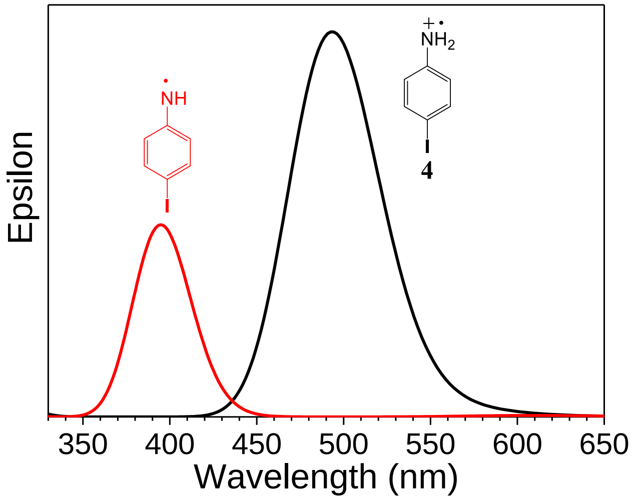

Supplementary Figure 9. Computed UV-vis spectra of the *p*-iodoanilino radical cation **4** (Black) and the *p*-iodo anilino radical (red) obtained by M06-2x calculations.

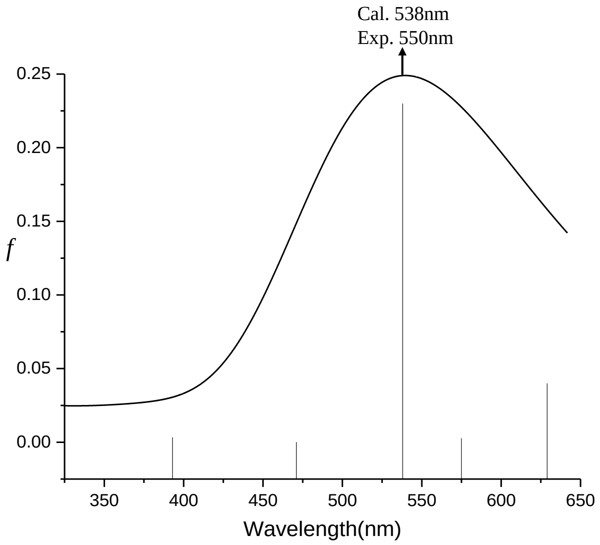

Supplementary Figure 10. The absorption spectra of triplet 4-iodophenylnitrenium ion ^{3}**2** (*np*) obtained at 8-roots-state-averaged CASPT2//CASSCF(10e/8o)/PCM level of theory.

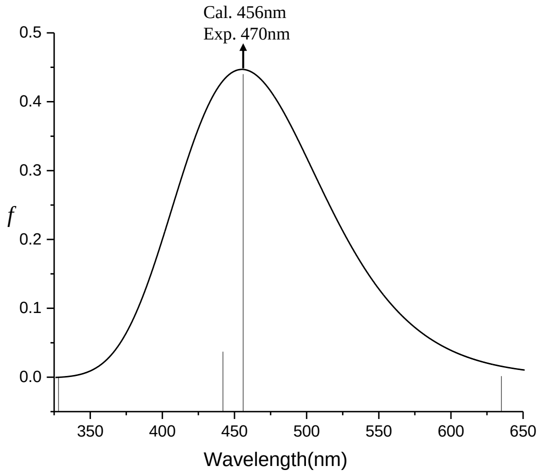

Supplementary Figure 11. The absorption spectra of closed-shell 4-iodophenylnitrenium ion ^{1}**2** (n^2) obtained at 5-roots-state-averaged CASPT2//CASSCF(10e/8o)/PCM level of theory.

Supplementary Table 1. Excitation energies ($\Delta E_{\perp}$, kcal/mol), oscillator strengths (*f*), wavelengths of absorption (λ_{ab}, nm) as well as the assignment of involved transitions of **^{3}2** (*np*) calculated at the 8-roots-state-averaged CASPT2//CASSCF(10e/8o)/PCM level of theory. The experimental values are given in parentheses.

	Transition	$\Delta E_{\perp}$	λ_{ab}	*f*	Singly occupied orbital
**^{3}2** (*np*)	$T_{NPz}(^3np)\rightarrow T_{PPz1}(^3\pi p)$	49.8	575	2.6E-03	n_1, $2p_z \rightarrow \pi_2$, $2p_z$
	$T_{PPz}(^3\pi p)\rightarrow T_{PP}(^3\pi\pi^*)$	45.5	629	4.0E-02	π_2, $2p_z \rightarrow \pi_1$, π_2^*
	$T_{NPz}(^3np)\rightarrow T_{NP1}(^3n\pi)$	53.2	**538(550)**	2.0E-01	n_1, $2p_z \rightarrow \pi_2$, n_1
	$T_{NPz}(^3np)\rightarrow T_{PPz2}(^3\pi p)$	60.7	471	1.9E-04	n_1, $2p_z \rightarrow \pi_1$, $2p_z$
	$T_{NPz}(^3np)\rightarrow T_{NP2}(^3n\pi)$	72.8	393	3.4E-03	n_1, $2p_z \rightarrow \pi_1$, n_1
	$T_{NPz}(^3np)\rightarrow T_{PP}(^3\pi\pi^*)$	95.2	300	2.1E-03	n_1, $2p_z \rightarrow \pi_1$, π_2^*
	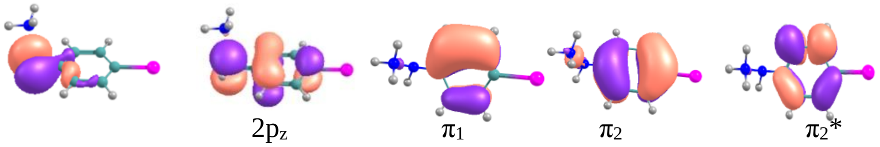				
	n_1	$2p_z$	π_1	π_2	π_2^*

Supplementary Table 2. Excitation energies ($\Delta E_{\perp}$, kcal/mol), oscillator strengths (*f*), wavelengths of absorption (λ_{ab}, nm) as well as the assignment of involved transitions of **^{1}2** (n^2) calculated at the 5-roots-state-averaged CASPT2//CASSCF(10e/8o)/PCM level of theory. The experimental values are given in parentheses.

	Transition	$\Delta E_{\perp}$	λ_{ab}	*f*	Singly occupied orbitals
**^{1}2** (n^2)	$S_0\rightarrow S_{NPz}(^1np)$	45.0	635	1.6E-03	n_1, $2p_z$
	$S_0\rightarrow S_{PPz1}(^1\pi p)$	62.8	**456(470)**	4.4E-01	$2p_z$, π_2
	$S_0\rightarrow S_{PPz2}(^1\pi p)$	64.7	442	3.7E-02	$2p_z$, π_1
	$S_0\rightarrow S_{NP}(^1n\pi)$	87.2	328	1.1E-04	n_1, π_1
	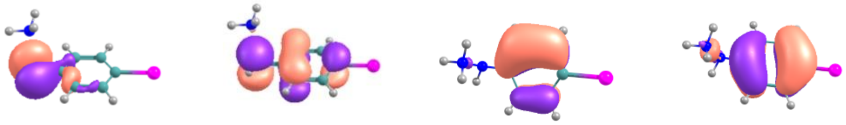				
	n_1	$2p_z$		π_1	π_2

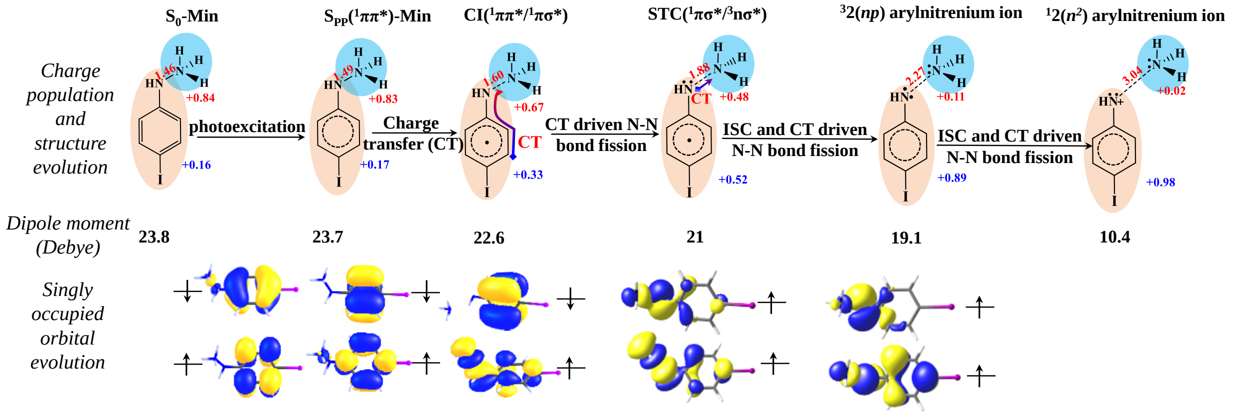

Supplementary Figure 12. Evolutions of the charge population, structure, dipole moment and singly occupied orbital along the critical points of the MEPs shown in Fig. 3 in the main alticle, which are calculated at the CASPT2//CASSCF/PCM/cc-pVDZ level of theory. Structures are given with their key bond lengths in Å.

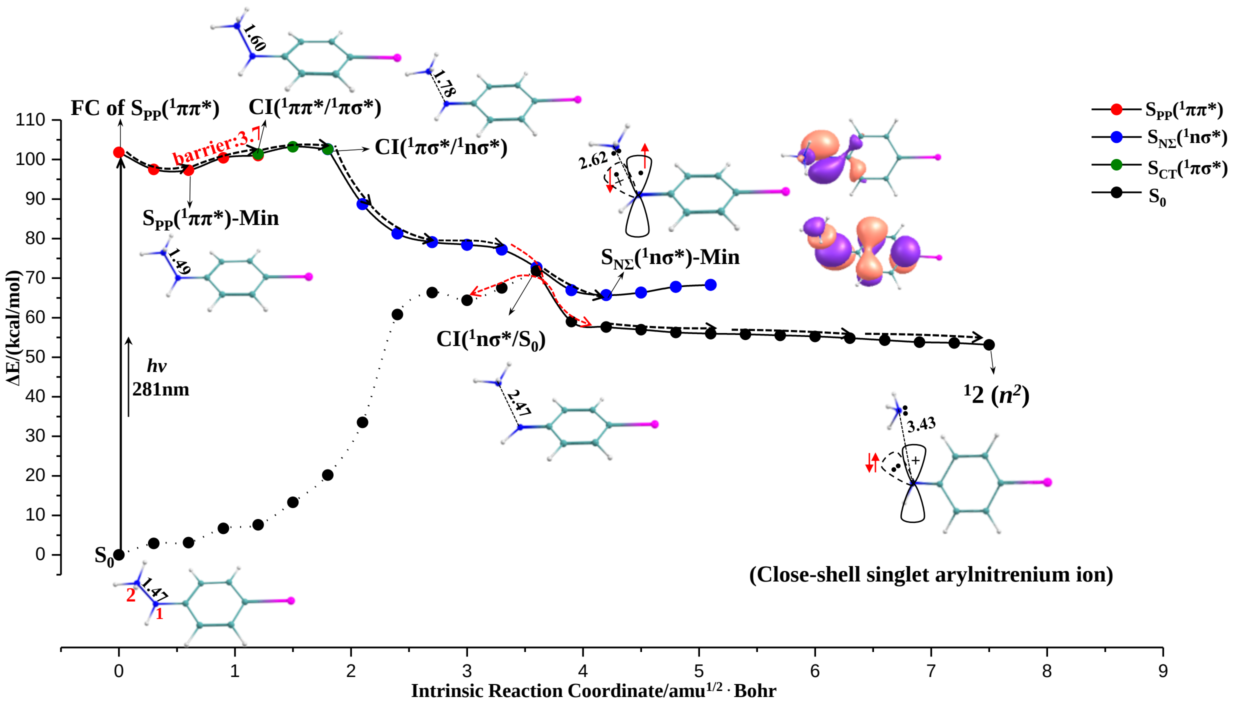

Supplementary Figure 13. Minimum energy profiles for the photolysis of **1** produing the close-shell singlet arylnitrenium ion of **^{1}2** (n^2) along the reaction channels of singlet states calculated at the CASPT2//CASSCF/PCM/cc-pVDZ level of theory. Selected stationary structures are given with their key bond lengths in Å while the singly occupied molecular orbitals for the selective singlet state are schematically provided.

Supplementary Table 3. Vertical excitation energies ($\Delta E_{\perp}$, kcal/mol), oscillator strengths (*f*), dipole moments (D.M., Debye), wavelengths of absorption (λ_{ab}, nm) as well as the assignment of involved transitions of **1** calculated at the 14-roots-state-averaged CASPT2//CASSCF(10e/8o)/PCM level of theory.

	Transition	$\Delta E_{\perp}$	λ_{ab}	D.M.	*f*	Singly occupied orbital*s*
**1**	S_0	--	--	23.6	--	--
	$S_0 \rightarrow S_{PP1}(^1\pi\pi^*)$	92.9	308	23.1	3.5E-02	π_1, π_1*
	$S_0 \rightarrow S_{PP2}(^1\pi\pi^*)$	126.3	226	21.0	5.6E-01	π_2, π_1*
	$S_0 \rightarrow S_{PP3}(^1\pi\pi^*)$	138.2	207	20.8	2.4E-02	π_2, π_2*
	$S_0 \rightarrow S_{CT1}(^1\pi\sigma^*)$	141.7	202	17.7	6.2E-01	π_1, σ*
	$S_0 \rightarrow S_{PP4}(^1\pi\pi^*)$	149.2	192	21.5	7.1E-01	π_1, π_2*
	$S_0 \rightarrow S_{CT2}(^1\pi\sigma^*)$	157.4	182	20.8	9.7E-02	π_2, σ*

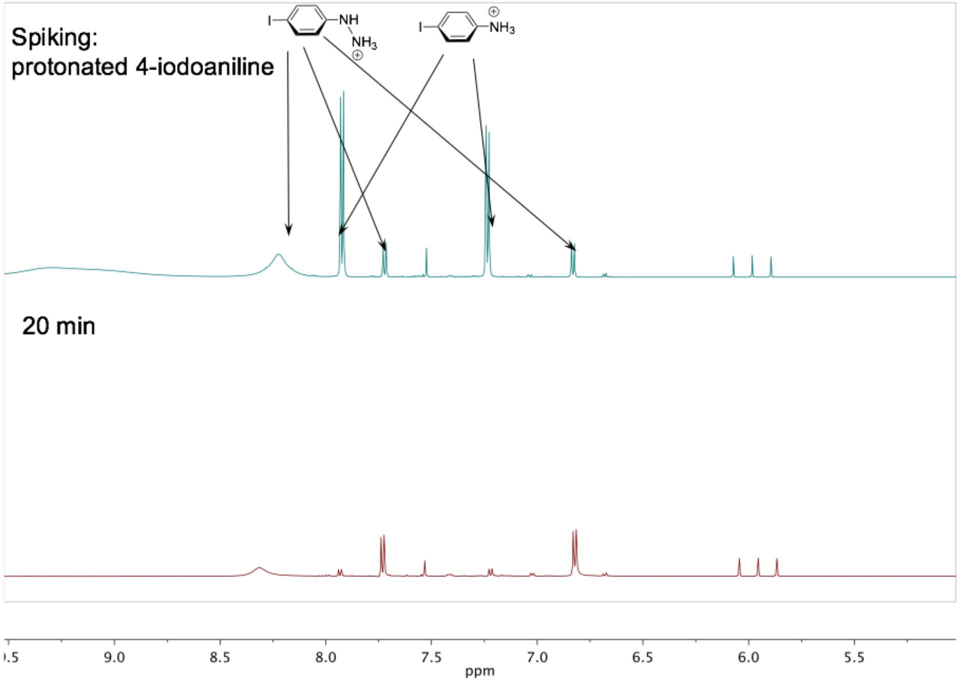

Supplementary Figure 14. ^{1}H NMR spectrum of **1** photolyzed for 20 min in regular acetonitrile and the assignment of peaks (top). The reduced protonated 4-iodoaniline was further confirmed by spiking experiments, where additional protonated 4-iodoaniline was added into the solution, and the two peaks assigned to this compound had grown higher (bottom).

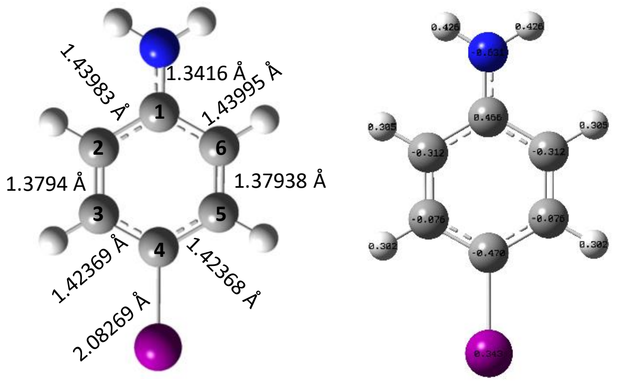

Supplementary Figure 15. **Optimized geometries of radical cation 4 obtained from the TD-M06-2X/LANL2DZcalculations.**The selected bond lengths (in Angstroms) (left), and Mulliken population analyses of radical cation **4** (right).

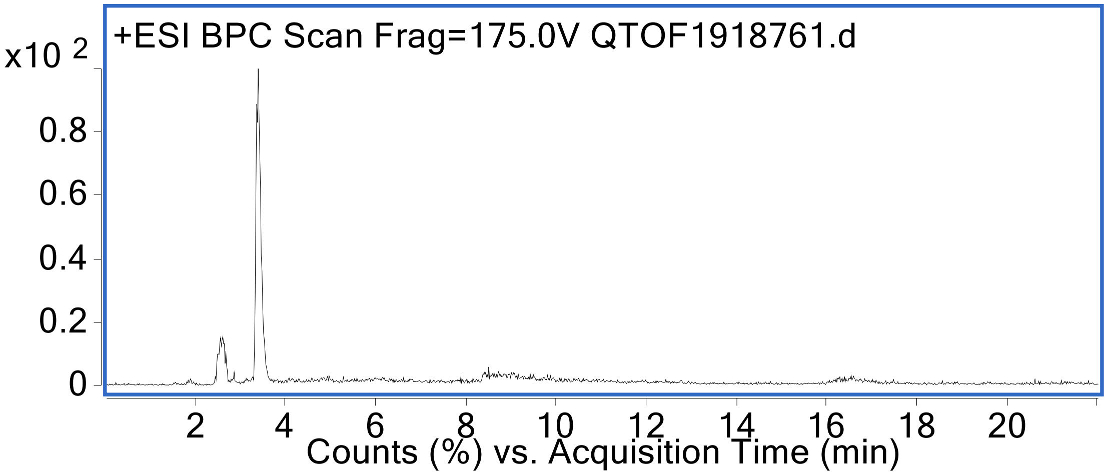

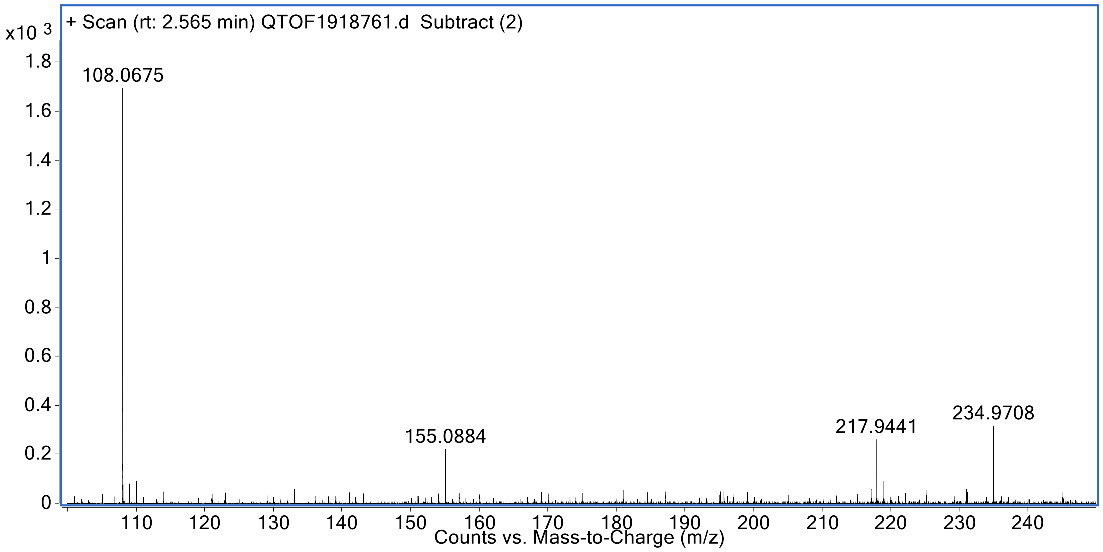

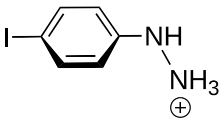

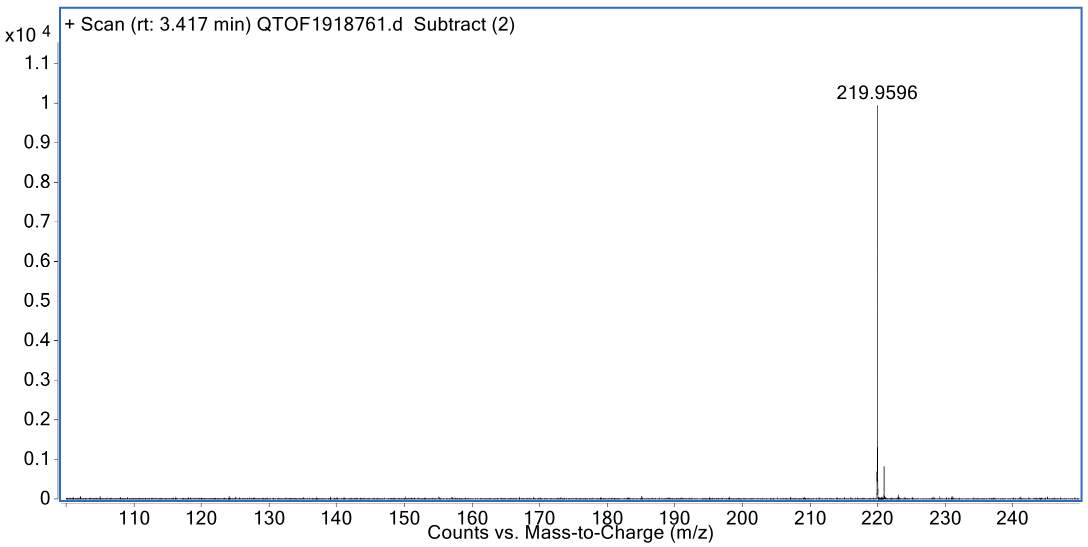

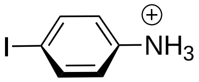

Supplementary Figure 16. LC-MS of **1** photolyzed for 30 min in regular acetonitrile.

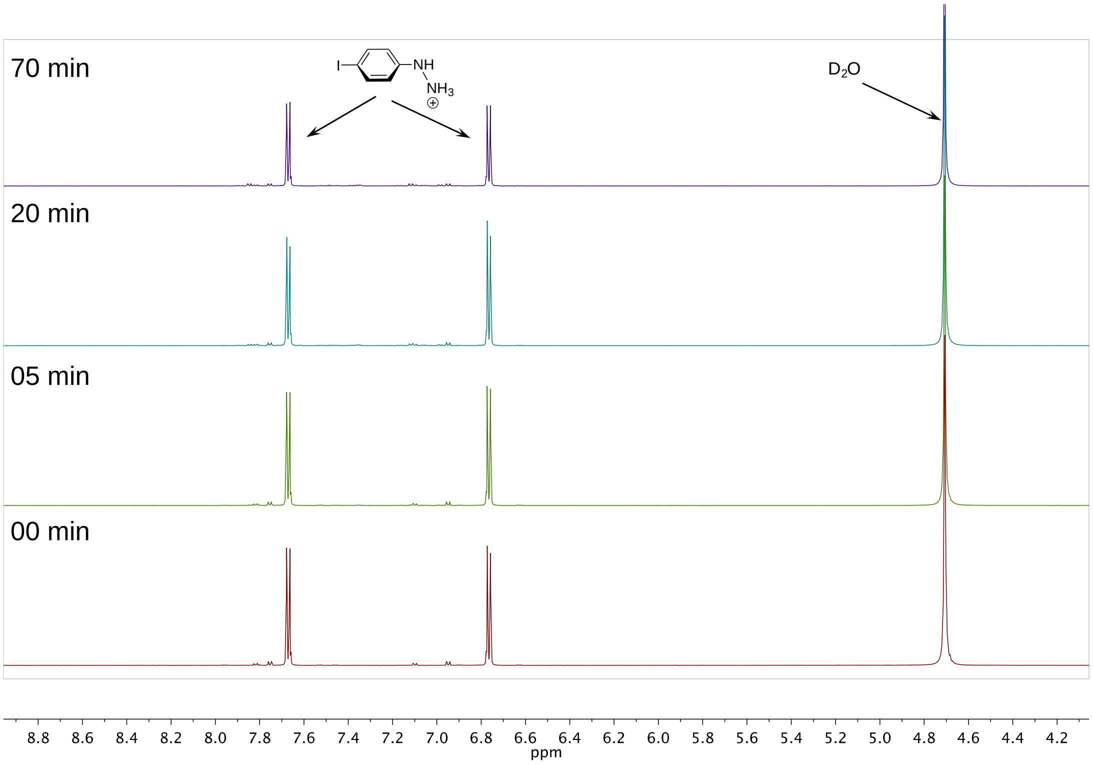

Supplementary Figure 17. ^{1}H NMR spectrum of **1** photolyzed for 20 min in water and the assignment of peaks (top). This photoprecusros appears to have deficient photo activity in water and exhibit no noticeable reaction over 70 minuets of irradiation, suggesting the photolysis of this precursor is highly solvent-dependent.

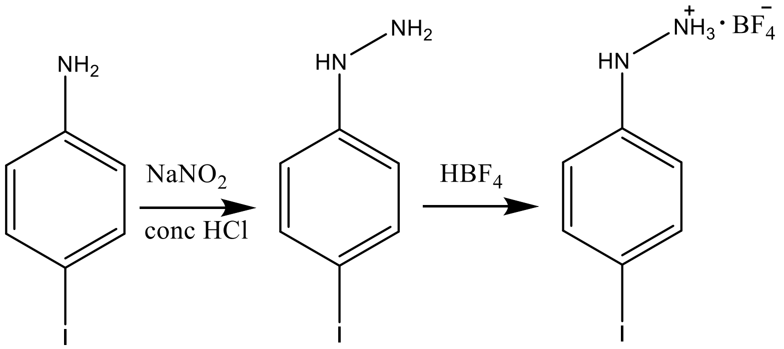

1

Supplementary Figure 18. **Synthesis route of 2-(4-iodo-phenyl)hydrazin-1-ium tetrafluoroborate 1. 4-iodoaniline (1.1 g 5 mmol) and 10 mL hydrochloric acid (5 M, 50 mmol) were in ice water bath at 0°C.** 4-iodoaniline (1.1 g 5 mmol) and 10 mL hydrochloric acid (5 M, 50 mmol) were in ice water bath at 0°C. Sodium nitrite (0.69 g, 10 mmol) dissolved in 1 mL DI water was added to the above mixture. Then the mixture was at 0°C for 1 hour. Stannous chloride dehydrate (3.3 g, 15 mmol) in 3 mL concentrated hydrochloric acid was prepared and dropped into the above solution and stirred for 2 hours. The brown mixture was basified with 40%

sodium hydroxide until the pH 10 and transferred to separating funnel to be extracted with ethyl acetate (3×50 mL) and washed with water(3×50mL). The brown-yellow solution was then dried with anhydrous sodium sulfate and treated with rotary evaporation to obtain the dark-yellow solid. The crude product was purified by column chromatography (75/25:EA/hexane) and the first two impurity bands were eluted. Finally, the product in the third band was collected by flash column chromatography using DCM. The solvent was removed by rotary evaporation to get the yellow solid, which is used directly without purification. The resulting product was dissolved in ether, to which HBF_4*OEt_2 was added until the TLC spot no longer moved, signifying the protonation of the hydrazine to the tetrafluoroborate salt. The precipitation was then filtered and dried under reduced pressure to yield the desired salt. The product was reasonably stable stored in a freezer in the dark. ^{1}H-NMR (600 MHz, D_2O): δ 7.67 (d, 2H), 6.77 (d, 2H); ^{13}C-NMR (600 MHz, MeCN-d_3): δ 143.43, 138.29, 117.73, 85.93; HRMS (ESI-QTOF) m/z: [M^+] calculated for $C_6H_8IN_2^+$, 234.9732; found 234.9728.

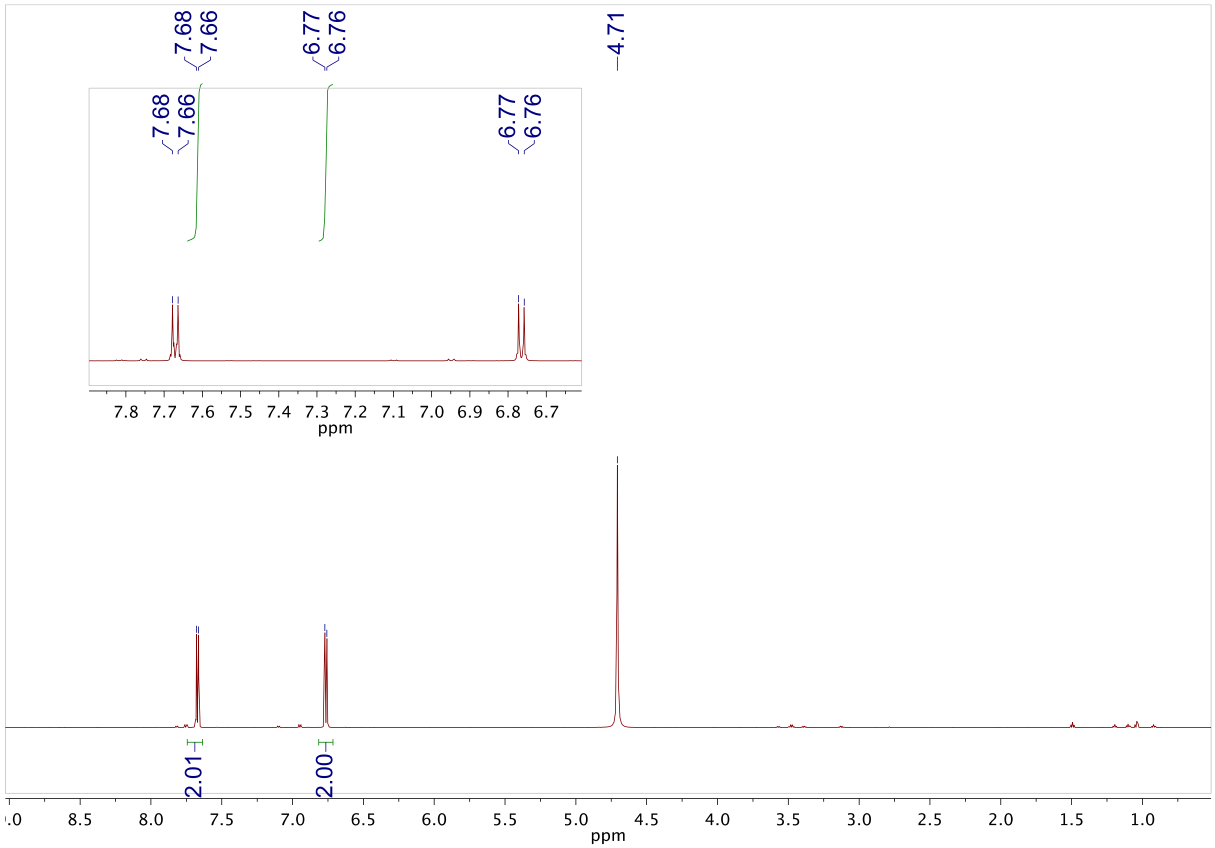

Supplementary Figure 19. ^{1}H NMR (600 MHz, D_2O) of 2-(4-iodo-phenyl)hydrazin-1-ium tetrafluoroborate **1**.

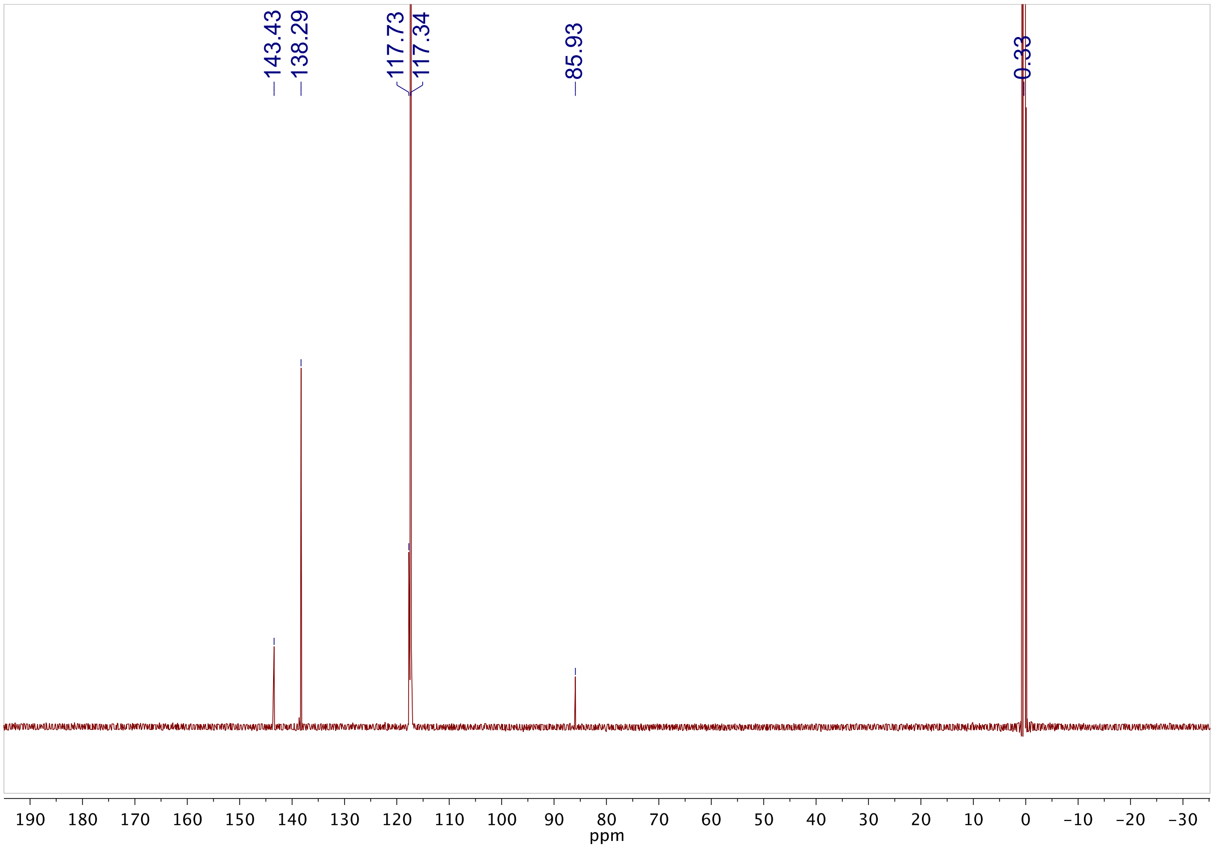

Supplementary Figure 20. ^{13}C NMR (MeCN-d$_3$) of 2-(4-iodo-phenyl)hydrazin-1-ium tetrafluoroborate **1**.

Supplementary Method

Computational details

Density functional theory (DFT) computations were performed using the Gaussian09 software suite. In all cases, optimized geometries were found to have zero imaginary frequencies and corrections for the zero-point vibrational energy were added unscaled. To predict the TR^3 spectra of the radical cation **4**, um062x/lanl2dz was employed to optimize the structure and predict the Raman spectra. A simulated band width for the spectra was determined using a Lorentzian of 10 cm^{-1} band width for the vibrational band frequencies. A frequency scaling factor of 0.960 was used in the comparison of the calculated results with the experimental spectra. Geometries of the radical cation **6** were optimized at the um062x/lanl2dz level of theory. TD-um062x/lanl2dz was utilized to simulate the UV−vis spectra of these two species. The electronic absorption spectra of the compounds were simulated by TD-DFT method, by solving the lowest 50 al-lowed vertical electronic transitions from the ground state. To simulate solvent broadening, each of the electronic transitions was represented by a Gaussian band shape with a half-width of 3500 cm^{-1}.

CASPT2//CASSCF Calculations

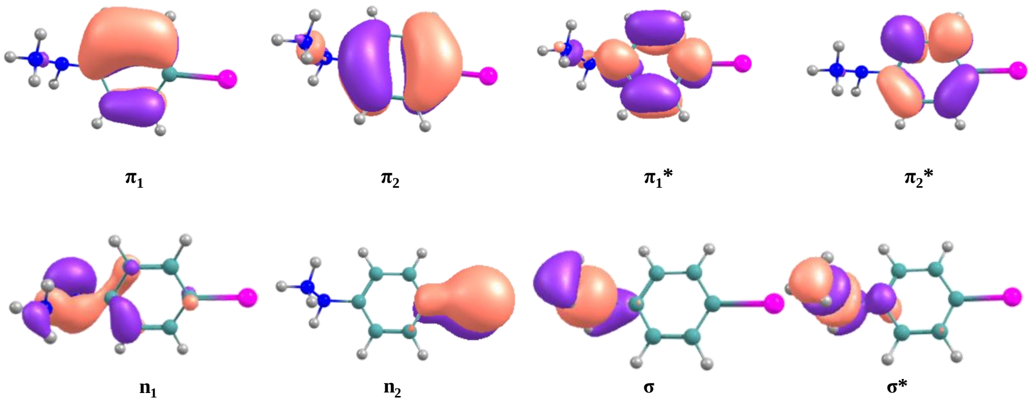

Supplementary Figure 21. Molecular orbitals of **1** used in defining the active space for the CASPT2//CASSCF (10e/8o) calculation.
