# Supplementary material for: Generation and direct observation of a triplet arylnitrenium ion
Source: Nat Commun. 2022 Jun 16;13:3458. doi: 10.1038/s41467-022-31091-z (PMC9203820; doi:10.1038/s41467-022-31091-z)
Supplement: Supplementary file 2 — Description of Additional Supplementary Files [file 41467_2022_31091_MOESM2_ESM.docx]

**Inventory of Supplementary Data File**

**Supplementary Data 1**. The absolute energies (A.E.) in Hartree and relative energies (R.E.) in kcal/mol along the minimum energy pathway for the photolysis of precursor **1** producing **^3^2** (*np*) and **^1^2** (*n^2^*) arylnitrenium ions through singlet and triplet state channels. The computational results were obtained at the CASPT2//CASSCF(10e/8o)/PCM/cc-pVDZ level of theory. The corresponding energy profiles are plotted in Fig. 3 of the main article………………………………………………………………………..1

**Supplementary Data 2**. The absolute energies (A.E.) in Hartree and relative energies (R.E.) in kcal/mol along the minimum energy pathway for the photolysis of precursor **1** producing **^1^2** (*n^2^*) arylnitrenium ions through singlet state channels. The computational results were obtained at the CASPT2//CASSCF(10e/8o)/PCM/cc-pVDZ level of theory. The corresponding energy profiles are plotted in Supplementary Figure 15 of the Supporting Information…………………………………….7
