# Supplementary material for: Generation and direct observation of a triplet arylnitrenium ion
Source: Nat Commun. 2022 Jun 16;13:3458. doi: 10.1038/s41467-022-31091-z (PMC9203820; doi:10.1038/s41467-022-31091-z)
Supplement: Supplementary file 3 — Supplementary Data 1 [file 41467_2022_31091_MOESM3_ESM.pdf]

Supplementary Data file

Table 1. The absolute energies (A.E.) in Hartree and relative energies (R.E.) in kcal/mol along the minimum energy pathway for the photolysis of precursor **1** producing ^{3}**2** (*np*) and ^{1}**2** (*n*^{2}) arylnitrenium ions through singlet and triplet state channels. The computational results were obtained at the CASPT2//CASSCF(10e/8o)/PCM/cc-pVDZ level of theory. The corresponding energy profiles are plotted in Fig. 3 of the main article.

		**RASSCF**	**CASPT2**	
		**A.E.**	**A.E.**	**R.E.**
**S_0-Min**	Root1(S_0)	-351.542067	-352.6043020162	0
	Root2[S_{PP}($^1\pi_1\pi_1$*)]	-351.331899	-352.4421200123	101.8
	Root3[S_{PP}($^1\pi_2\pi_1$*)]	-351.274011		
	Root4[S_{CT}($^1n_1\pi_1$*)]	-351.236742		
	Root5[S_{PP}($^1\pi_2\pi_2$*)]	-351.223143		
Path-S_{PP}($^1\pi\pi$*)-1	Root1(S_0)	-351.533103	-352.5996298418	2.9
	Root2[S_{PP}($^1\pi_1\pi_1$*)]	-351.344425	-352.4488645322	97.5
	Root3	-351.275828		
	Root4	-351.239185		
	Root5	-351.231142		
**S_{PP}($^1\pi\pi$*)-Min**	Root1(S_0)	-351.532848	-352.5993898989	3.1
	Root2[S_{PP}($^1\pi_1\pi_1$*)]	-351.345300	-352.4492817425	97.3
	Root3[S_{PP}($^1\pi_2\pi_1$*)]	-351.275841		
	Root4[S_{CT}($^1n_1\pi_1$*)]	-351.240205		
	Root5[S_{PP}($^1\pi_1\pi_2$*)]	-351.232237		
Path-S_{PP}($^1\pi\pi$*)-3	Root1(S_0)	-351.526951	-352.5936634211	6.7
	Root2[S_{PP}($^1\pi_1\pi_1$*)]	-351.341200	-352.4443446965	100.4
	Root3	-351.281614		
	Root4	-351.256439		
	Root5	-351.232919		
	(10e/8o)			
	Root1(S_0)	-351.525156	-352.5921576457	7.6
	Root2[S_{PP}($^1\pi_1\pi_1$*)]	-351.340017	-352.4433713842	101.0

**CI($^1\pi\pi^*/^1\pi\sigma^*$)**	Root3[$S_{PP}(^1\pi_2\pi_1^*)$]	-351.286720		
	Root4[$S_{CT}(^1n_1\pi_1^*)$]	-351.258411		
	Root5[$S_{PP}(^1\pi_2\pi_2^*)$]	-351.233871		
	(6e/6o)			
	Root1(S_0)	-351.832386	-352.9240293162	
	Root2[$S_{PP}(^1\pi_1\pi_1^*)$]	-351.630235	-352.7800900260	
	Root3[$S_{PP}(^1\pi_2\pi_1^*)$]	-351.569041		
	Root4[$S_{PP}(^1\pi_2\pi_2^*)$]	-351.518616		
	Root5[$S_{CT}(^1\pi_2\sigma^*)$]	-351.505529		
	Root6[$S_{PP}(^1\pi_1\pi_2^*)$]	-351.492654		
	Root7[$S_{CT}(^1\pi_1\sigma^*)$]	-351.460358	-352.7793424073	
Path-$S_{CT}(^1\pi\sigma^*)$-1	Root1(S_0)	-351.515430	-352.5831041436	13.3
	Root2[$S_{CT}(^1\pi_1\sigma^*)$]	-351.334939	-352.4397847335	103.2
	Root3	-351.313078		
	Root4	-351.267992		
	Root5	-351.246313		
Path-$S_{CT}(^1\pi\sigma^*)$-2	Root1(S_0)	-351.503059	-352.5721660053	20.2
	Root2[$S_{CT}(^1\pi_1\sigma^*)$]	-351.330441	-352.4411158565	102.4
	Root3	-351.327459		
	Root4	-351.283188		
	Root5	-351.248291		
**STC($^1\pi\sigma^*/^3n\sigma^*$)**	Root1(S_0)	-351.489071	-352.5586776691	28.6
	Root2[$S_{CT}(^1\pi_2\sigma^*)$]	-351.336046		
	Root3[$S_{CT}(^1\pi_1\sigma^*)$]	-351.327751	-352.4402819479	102.9
	Root4[$S_{N\Sigma}(^1n_1\sigma^*)$]	-351.291711		
	Root5[double excitation($\pi_2\rightarrow\sigma^*$)]	-351.256368		
	Root1[$T_{CT}(^3\pi_2\sigma^*)$]	-351.398173		
	Root2[$T_{CT}(^3\pi_1\sigma^*)$]	-351.344676		
	Root3[$T_{N\Sigma}(^3n_1\sigma^*)$]	-351.338299	-352.4400257246	103.1

	Root4[T_{PP}($^3\pi_1\pi_2$*)]	-351.326476		
	Root5[T_{CT}($^3\sigma\sigma$*)]	-351.281479		
Path-$T_{N\Sigma}$($^3n\sigma$*)-1	Root1	-351.422125		
	Root2[$T_{N\Sigma}$($^3n_1\sigma$*)]	-351.356078	-352.4537239975	94.5
	Root3	-351.339652		
	Root4	-351.317493		
	Root5	-351.303055		
	Root1(S_0)	-351.489462	-352.5556288357	30.5
	Root2	-351.367207		
	Root3	-351.329974		
	Root4	-351.314131		
	Root5	-351.286027		
Path-$T_{N\Sigma}$($^3n\sigma$*)-2	Root1	-351.394898		
	Root2[$T_{N\Sigma}$($^3n_1\sigma$*)]	-351.356658	-352.4609868701	89.9
	Root3	-351.341505		
	Root4	-351.304854		
	Root5	-351.292660		
	Root1(S_0)	-351.462948	-352.5410329729	39.7
	Root2	-351.340545		
	Root3	-351.337723		
	Root4	-351.320117		
	Root5	-351.277456		
Path-$T_{N\Sigma}$($^3n\sigma$*)-3	Root1[$T_{N\Sigma}$($^3n_1\sigma$*)]	-351.433370	-352.5134657043	57.0
	Root2	-351.355695		
	Root3	-351.342399		
	Root4	-351.318725		
	Root5	-351.314224		

	Root1(S_0)	-351.460135	-352.5331991494	44.6
	Root2	-351.385260		
	Root3	-351.336915		
	Root4	-351.323358		
	Root5	-351.319391		
	Root1[$T_{N\Sigma}$($^3n_1\sigma^*$)]	-351.438933	-352.5181602178	54.1
	Root2[T_{CT}($^3\pi_2\sigma^*$)]	-351.357151		
	Root3[T_{CT}($^3\pi_1\sigma^*$)]	-351.341294		
	Root4[T_{NP}($^3n_1\pi_1$)]	-351.328453		
	Root5[T_{NP}($^3n_1\pi_2$)]	-351.326194		
**^{3}2** (*np*)				
	Root1(S_0)	-351.451445	-352.5140803442	56.6
	Root2[$S_{N\Sigma}$($^1n_1\sigma^*$)]	-351.399706		
	Root3[S_{CT}($^1\pi_1\sigma^*$)]	-351.341163		
	Root4[S_{NP}($^1n_1\pi_1$)]	-351.327874		
	Root5[S_{NP}($^1n_1\pi_2$)]	-351.314159		
	Root1[$T_{N\Sigma}$($^3n_1\sigma^*$)]	-351.435858	-352.5114121337	58.3
	Root2[T_{CT}($^3\pi_2\sigma^*$)]	-351.374140		
	Root3[T_{CT}($^3\pi_1\sigma^*$)]	-351.355338		
	Root4[T_{NP}($^3n_1\pi_1$)]	-351.347782		
	Root5[T_{NP}($^3n_1\pi_2$)]	-351.312689		
**STC($^3n\sigma^*/S_0$)**				
	Root1(S_0)	-351.454892	-352.5100607723	59.2
	Root2[$S_{N\Sigma}$($^1n_1\sigma^*$)]	-351.415225		
	Root3[S_{CT}($^1\pi_1\sigma^*$)]	-351.359209		
	Root4[S_{NP}($^1n_1\pi_1$)]	-351.349641		
	Root5[S_{CT}($^1\pi_2\sigma^*$)]	-351.297385		
	Root1[$T_{N\Sigma}$($^3n_1\sigma^*$)]	-351.442168	-352.5104876664	58.9
	Root2	-351.379373		
Path-$T_{N\Sigma}$($^3n\sigma^*$)-6	Root3	-351.362183		

	Root4	-351.355433		
	Root5	-351.312841		
Path-$T_{N\Sigma}(^3n\sigma^*)$-7	Root1[$T_{N\Sigma}(^3n_1\sigma^*)$]	-351.447949	-352.5113524409	58.3
	Root2	-351.382069		
	Root3	-351.370393		
	Root4	-351.357576		
	Root5	-351.316525		
Path- S_0-1	Root1(S_0)	-351.468881	-352.5196339481	53.1
	Root2	-351.390973		
	Root3	-351.364953		
	Root4	-351.318765		
	Root5	-351.307669		
Path- S_0-2	Root1(S_0)	-351.470196	-352.5196524443	53.1
	Root2	-351.393886		
	Root3	-351.367036		
	Root4	-351.322310		
	Root5	-351.309848		
Path- S_0-3	Root1(S_0)	-351.471207	-352.5196296519	53.1
	Root2	-351.396218		
	Root3	-351.368784		
	Root4	-351.325525		
	Root5	-351.311577		
Path- S_0-4	Root1(S_0)	-351.471687	-352.5191810150	53.4
	Root2	-351.398129		
	Root3	-351.369871		
	Root4	-351.328223		
	Root5	-351.312732		
Path- S_0-5	Root1(S_0)	-351.472018	-352.5191187740	53.4
	Root2	-351.399396		
	Root3	-351.370866		

	Root4	-351.330256		
	Root5	-351.313829		
	Root1(S_0)	-351.472525	-352.5188887837	53.4
	Root2[$S_{N\Sigma}$($^1n_1\sigma^*$)]	-351.400611		
^{1}**2** (n^2)	Root3[S_{CT}($^1\pi_1\sigma^*$)]	-351.371817		
	Root4[S_{NP}($^1n_1\pi_1$)]	-351.332217		
	Root5[S_{CT}($^1\pi_2\sigma^*$)]	-351.315138		

Table 2. The absolute energies (A.E.) in Hartree and relative energies (R.E.) in kcal/mol along the minimum energy pathway for the photolysis of precursor **1** producing **^{1}2** (n^2) arylnitrenium ions through singlet state channels. The computational results were obtained at the CASPT2//CASSCF(10e/8o)/PCM/cc-pVDZ level of theory. The corresponding energy profiles are plotted in Supplementary Figure 15 of the Supporting Information.

		**RASSCF**	**CASPT2**	
		**A.E.**	**A.E.**	**R.E.**
**S_0-Min**	Root1(S_0)	-351.542067	-352.6043020162	0
	Root2[S_{PP}($^1\pi_1\pi_1$*)]	-351.331899	-352.4421200123	101.8
	Root3[S_{PP}($^1\pi_2\pi_1$*)]	-351.274011		
	Root4[S_{CT}($^1n_1\pi_1$*)]	-351.236742		
	Root5[S_{PP}($^1\pi_2\pi_2$*)]	-351.223143		
Path-S_{PP}($^1\pi\pi$*)-1	Root1(S_0)	-351.533103	-352.5996298418	2.9
	Root2[S_{PP}($^1\pi_1\pi_1$*)]	-351.344425	-352.4488645322	97.5
	Root3	-351.275828		
	Root4	-351.239185		
	Root5	-351.231142		
**S_{PP}($^1\pi\pi$*)-Min**	Root1(S_0)	-351.532848	-352.5993898989	3.1
	Root2[S_{PP}($^1\pi_1\pi_1$*)]	-351.345300	-352.4492817425	97.3
	Root3[S_{PP}($^1\pi_2\pi_1$*)]	-351.275841		
	Root4[S_{CT}($^1n_1\pi_1$*)]	-351.240205		
	Root5[S_{PP}($^1\pi_1\pi_2$*)]	-351.232237		
Path-S_{PP}($^1\pi\pi$*)-3	Root1(S_0)	-351.526951	-352.5936634211	6.7
	Root2[S_{PP}($^1\pi_1\pi_1$*)]	-351.341200	-352.4443446965	100.4
	Root3	-351.281614		
	Root4	-351.256439		
	Root5	-351.232919		
	(10e/8o)			
	Root1(S_0)	-351.525156	-352.5921576457	7.6
	Root2[S_{PP}($^1\pi_1\pi_1$*)]	-351.340017	-352.4433713842	101.0
	Root3[S_{PP}($^1\pi_2\pi_1$*)]	-351.286720		
	Root4[S_{CT}($^1n_1\pi_1$*)]	-351.258411		

	Root5[$S_{PP}(^1\pi_2\pi_2^*)$]	-351.233871		
**CI($^1\pi\pi^*/^1\pi\sigma^*$)**	(6e/6o)			
	Root1(S_0)	-351.832386	-352.9240293162	
	Root2[$S_{PP}(^1\pi_1\pi_1^*)$]	-351.630235	-352.7800900260	
	Root3[$S_{PP}(^1\pi_2\pi_1^*)$]	-351.569041		
	Root4[$S_{PP}(^1\pi_2\pi_2^*)$]	-351.518616		
	Root5[$S_{CT}(^1\pi_2\sigma^*)$]	-351.505529		
	Root6[$S_{PP}(^1\pi_1\pi_2^*)$]	-351.492654		
	Root7[$S_{CT}(^1\pi_1\sigma^*)$]	-351.460358	-352.7793424073	
Path-$S_{CT}(^1\pi\sigma^*)$-1	Root1(S_0)	-351.515430	-352.5831041436	13.3
	Root2[$S_{CT}(^1\pi_1\sigma^*)$]	-351.334939	-352.4397847335	103.2
	Root3	-351.313078		
	Root4	-351.267992		
	Root5	-351.246313		
**CI($^1\pi\sigma^*/^1n\sigma^*$)**	Root1(S_0)	-351.503632	-352.5730159998	19.6
	Root2[$S_{CT}(^1\pi_1\sigma^*)$]	-351.330743	-352.4407562779	102.6
	Root3[$S_{CT}(^1n_1\sigma^*)$]	-351.328035	-352.4408367207	102.6
	Root4[double excitation($\pi_2\rightarrow\sigma^*$)]	-351.284247		
	Root5[$S_{CT}(^1\pi_2\sigma^*)$]	-351.250355		
Path-$S_{N\Sigma}(^1n\sigma^*)$-1	Root1(S_0)	-351.490312	-352.5509644183	33.5
	Root2[$S_{N\Sigma}(^1n_1\sigma^*)$]	-351.386479	-352.4629926595	88.7
	Root3	-351.314443		
	Root4	-351.307718		
	Root5	-351.264007		
Path-$S_{N\Sigma}(^1n\sigma^*)$-2	Root1(S_0)	-351.445856	-352.5074107647	60.8
	Root2[$S_{N\Sigma}(^1n_1\sigma^*)$]	-351.411808	-352.4747872115	81.3
	Root3	-351.308617		
	Root4	-351.292791		
	Root5	-351.245315		
	Root1(S_0)	-351.436342	-352.4986071101	66.3

Path-$S_{N\Sigma}$($^1n\sigma^*$)-3	Root2[$S_{N\Sigma}$($^1n_1\sigma^*$)]	-351.420224	-352.4782873000	79.1
	Root3	-351.303476		
	Root4	-351.288424		
	Root5	-351.265082		
Path-$S_{N\Sigma}$($^1n\sigma^*$)-4	Root1(S_0)	-351.426128	-352.5017125483	64.4
	Root2[$S_{N\Sigma}$($^1n_1\sigma^*$)]	-351.396980	-352.4793170353	78.4
	Root3	-351.335938		
	Root4	-351.317107		
	Root5	-351.304806		
Path-$S_{N\Sigma}$($^1n\sigma^*$)-5	Root1(S_0)	-351.430229	-352.4967022440	67.5
	Root2[$S_{N\Sigma}$($^1n_1\sigma^*$)]	-351.420749	-352.4812791867	77.2
	Root3	-351.353089		
	Root4	-351.325082		
	Root5	-351.300298		
**CI($^1n\sigma^*/S_0$)**	Root1(S_0)	-351.431861	-352.4899089733	71.8
	Root2[$S_{N\Sigma}$($^1n_1\sigma^*$)]	-351.428673	-352.4884683547	72.7
	Root3[S_{NP}($^1n_1\pi_1$)]	-351.357216		
	Root4[S_{CT}($^1\pi_1\sigma^*$)]	-351.330670		
	Root5[S_{NP}($^1n_1\pi_2$)]	-351.303874		
Path-$S_{N\Sigma}$($^1n\sigma^*$)-7	Root1[$S_{N\Sigma}$($^1n_1\sigma^*$)]	-351.439982	-352.4976023365	67.0
	Root2(S_0)	-351.433975		
	Root3	-351.366924		
	Root4	-351.330948		
	Root5	-351.312525		
**$S_{N\Sigma}$($^1n\sigma^*$)-Min**	Root1[$S_{N\Sigma}$($^1n_1\sigma^*$)]	-351.442907	-352.4995786206	65.7
	Root2(S_0)	-351.436645		
	Root3	-351.368510		
	Root4	-351.331260		
	Root5	-351.314627		
	Root1[$S_{N\Sigma}$($^1n_1\sigma^*$)]	-351.444044	-352.4986008484	66.3

Path-$S_{N\Sigma}$($^1n\sigma^*$)-9	Root2(S_0)	-351.438780		
	Root3	-351.370358		
	Root4	-351.333816		
	Root5	-351.316347		
Path-$S_{N\Sigma}$($^1n\sigma^*$)-10	Root1(S_0)	-351.446875		
	Root2[$S_{N\Sigma}$($^1n_1\sigma^*$)]	-351.441277	-352.4962797137	67.8
	Root3	-351.369458		
	Root4	-351.344016		
	Root5	-351.314905		
Path-$S_{N\Sigma}$($^1n\sigma^*$)-11	Root1(S_0)	-351.449512	-352.5007157128	65.0
	Root2[$S_{N\Sigma}$($^1n_1\sigma^*$)]	-351.441394	-352.4954676132	68.3
	Root3	-351.369815		
	Root4	-351.347145		
	Root5	-351.315163		
Path- S_0-1	Root1(S_0)	-351.458944	-352.5102012214	59.0
	Root2	-351.399237		
	Root3	-351.357006		
	Root4	-351.323568		
	Root5	-351.300318		
Path- S_0-2	Root1(S_0)	-351.463455	-352.5124552581	57.6
	Root2	-351.398569		
	Root3	-351.361873		
	Root4	-351.325394		
	Root5	-351.304692		
Path- S_0-3	Root1(S_0)	-351.465830	-352.5135167498	57.0
	Root2	-351.399392		
	Root3	-351.364404		
	Root4	-351.328272		
	Root5	-351.307079		
	Root1(S_0)	-351.467662	-352.5146018178	56.3

Path- S_0-4	Root2	-351.399931		
	Root3	-351.366638		
	Root4	-351.330361		
	Root5	-351.309524		
Path- S_0-5	Root1(S_0)	-351.468401	-352.5151348607	56.0
	Root2	-351.400093		
	Root3	-351.368081		
	Root4	-351.332606		
	Root5	-351.312333		
Path- S_0-6	Root1(S_0)	-351.468789	-352.5154092158	55.8
	Root2	-351.400160		
	Root3	-351.368515		
	Root4	-351.332746		
	Root5	-351.312665		
Path- S_0-7	Root1(S_0)	-351.469048	-352.5158527403	55.5
	Root2	-351.400197		
	Root3	-351.368928		
	Root4	-351.332937		
	Root5	-351.312939		
Path- S_0-8	Root1(S_0)	-351.469461	-352.5162635372	55.2
	Root2	-351.400513		
	Root3	-351.369345		
	Root4	-351.333282		
	Root5	-351.313236		
Path- S_0-9	Root1(S_0)	-351.469994	-352.5169447590	54.8
	Root2	-351.400731		
	Root3	-351.369999		
	Root4	-351.333666		
	Root5	-351.313711		
	Root1(S_0)	-351.470638	-352.5177473505	54.3

Path- S_0-10	Root2	-351.401050		
	Root3	-351.370697		
	Root4	-351.334101		
	Root5	-351.314229		
Path- S_0-11	Root1(S_0)	-351.471331	-352.5185576896	53.8
	Root2	-351.401406		
	Root3	-351.371364		
	Root4	-351.334527		
	Root5	-351.314749		
Path- S_0-12	Root1(S_0)	-351.471657	-352.5189226337	53.6
	Root2	-351.401533		
	Root3	-351.371733		
	Root4	-351.334765		
	Root5	-351.314988		
^{1}**2** (n^2)	Root1(S_0)	-351.472372	-352.5196338644	53.1
	Root2[$S_{N\Sigma}(^1n_1\sigma^*)$]	-351.401922		
	Root3[$S_{CT}(^1\pi_1\sigma^*)$]	-351.372374		
	Root4[$S_{N\Sigma}(^1n_1\sigma^*)$]	-351.335281		
	Root5[$S_{CT}(^1\pi_2\sigma^*)$]	-351.315547		
