# Supplementary material for: Generation and direct observation of a triplet arylnitrenium ion
Source: Nat Commun. 2022 Jun 16;13:3458. doi: 10.1038/s41467-022-31091-z (PMC9203820; doi:10.1038/s41467-022-31091-z)
Supplement: Supplementary file 4 — Supplementary Data 2 [file 41467_2022_31091_MOESM4_ESM.pdf]

**Cartesian Coordinates:**

**$S_0$-Min**

| | | | |
|---|---|---|---|
| C | -0.446706000 | -1.190459000 | -0.134655000 |
| C | -1.822443000 | -1.232329000 | -0.241008000 |
| C | -2.554363000 | -0.050439000 | -0.342353000 |
| C | -1.899432000 | 1.178509000 | -0.345765000 |
| C | -0.511252000 | 1.227002000 | -0.218215000 |
| C | 0.208491000 | 0.038489000 | -0.116247000 |
| I | 2.304101000 | 0.098451000 | 0.062001000 |
| N | -3.980644000 | -0.060523000 | -0.489539000 |
| N | -4.656955000 | 0.131666000 | 0.808181000 |
| H | 0.108763000 | -2.114735000 | -0.062881000 |
| H | -2.326921000 | -2.191265000 | -0.251454000 |
| H | -2.466923000 | 2.093477000 | -0.456663000 |
| H | -0.006627000 | 2.182284000 | -0.215401000 |
| H | -4.316854000 | -0.952522000 | -0.824985000 |
| H | -5.660782000 | 0.140655000 | 0.637999000 |
| H | -4.439881000 | -0.588275000 | 1.497233000 |
| H | -4.402293000 | 1.038775000 | 1.189635000 |

**$S_{PP}(^1\pi\pi^*)$-Min**

| | | | |
|---|---|---|---|
| C | -0.477492000 | -1.200135000 | -0.140994000 |
| C | -1.850434000 | -1.239766000 | -0.247470000 |
| C | -2.626877000 | -0.060258000 | -0.356036000 |
| C | -1.976085000 | 1.238357000 | -0.360724000 |
| C | -0.513973000 | 1.294344000 | -0.218856000 |
| C | 0.221418000 | 0.038938000 | -0.115486000 |
| I | 2.294793000 | 0.081764000 | 0.059685000 |
| N | -4.037982000 | -0.096940000 | -0.484914000 |
| N | -4.716550000 | 0.139365000 | 0.815834000 |
| H | 0.069627000 | -2.128704000 | -0.073856000 |
| H | -2.345941000 | -2.202662000 | -0.259161000 |
| H | -2.545465000 | 2.140579000 | -0.520263000 |

| | | | |
|---|---|---|---|
| H | -0.007187000 | 2.244469000 | -0.216836000 |
| H | -4.369402000 | -1.005041000 | -0.779837000 |
| H | -5.722225000 | 0.114128000 | 0.659291000 |
| H | -4.474303000 | -0.538088000 | 1.538661000 |
| H | -4.481453000 | 1.071391000 | 1.146800000 |

**CI($^1\pi\pi^*/^1\pi\sigma^*$)**

| | | | |
|---|---|---|---|
| C | -0.457235180 | -1.210046510 | -0.134078220 |
| C | -1.828772180 | -1.243901510 | -0.257509220 |
| C | -2.600165180 | -0.052563510 | -0.397293220 |
| C | -1.934122180 | 1.240798490 | -0.367373220 |
| C | -0.471830180 | 1.276454490 | -0.223998220 |
| C | 0.246803820 | 0.016916490 | -0.115662220 |
| I | 2.321310820 | 0.044577490 | 0.066187780 |
| N | -4.000614180 | -0.071252510 | -0.536086220 |
| N | -4.720731820 | 0.124491510 | 0.877385220 |
| H | 0.083103820 | -2.141984510 | -0.051073220 |
| H | -2.331326180 | -2.202957510 | -0.270651220 |
| H | -2.495040180 | 2.150160490 | -0.513812220 |
| H | 0.046899820 | 2.220713490 | -0.219100220 |
| H | -4.337128180 | -0.984591510 | -0.810584220 |
| H | -5.722545820 | 0.073756510 | 0.710826220 |
| H | -4.446736820 | -0.571586490 | 1.567486220 |
| H | -4.495349820 | 1.055145510 | 1.215682220 |

**STC($^1\pi\sigma^*/^3n\sigma^*$)**

| | | | |
|---|---|---|---|
| C | -0.385940000 | -1.201782000 | -0.148706000 |
| C | -1.817007000 | -1.255732000 | -0.307663000 |
| C | -2.615039000 | -0.083044000 | -0.464878000 |
| C | -1.908041000 | 1.152698000 | -0.399829000 |
| C | -0.478438000 | 1.214756000 | -0.208838000 |
| C | 0.283917000 | 0.032643000 | -0.095951000 |
| I | 2.364595000 | 0.122762000 | 0.144768000 |
| N | -3.932489000 | -0.081299000 | -0.642567000 |

| | | | |
|---|---|---|---|
| N | -4.794819000 | 0.180625000 | 1.002311000 |
| H | 0.153711000 | -2.134653000 | -0.062547000 |
| H | -2.300351000 | -2.225396000 | -0.332226000 |
| H | -2.465358000 | 2.077392000 | -0.490306000 |
| H | -0.012431000 | 2.190189000 | -0.170179000 |
| H | -4.289238000 | -1.011768000 | -0.768124000 |
| H | -5.775867000 | 0.130150000 | 0.781035000 |
| H | -4.552458000 | -0.509704000 | 1.696334000 |
| H | -4.556108000 | 1.107973000 | 1.311451000 |

$^{3}$**2** (*np*)

| | | | |
|---|---|---|---|
| 6 | -0.452611651 | -1.198344751 | -0.100717052 |
| 6 | -1.797399846 | -1.251012752 | -0.334880085 |
| 6 | -2.507835950 | -0.047307584 | -0.550398114 |
| 6 | -1.867917858 | 1.200010596 | -0.478697105 |
| 6 | -0.495665660 | 1.231114598 | -0.255932073 |
| 6 | 0.214285443 | 0.042106427 | -0.073041044 |
| 53 | 2.287442744 | 0.136410443 | 0.245162999 |
| 7 | -3.833633142 | -0.112383595 | -0.771630146 |
| 7 | -4.901486118 | 0.156175595 | 1.215260209 |
| 1 | 0.091295426 | -2.118270879 | 0.057771968 |
| 1 | -2.316751924 | -2.199431894 | -0.360743089 |
| 1 | -2.427928937 | 2.113185723 | -0.621403128 |
| 1 | 0.008222414 | 2.184758735 | -0.228678069 |
| 1 | -4.502437232 | -0.747211680 | -1.207634209 |
| 1 | -5.904008262 | 0.227178608 | 1.119367201 |
| 1 | -4.727536095 | -0.609136513 | 1.850811303 |
| 1 | -4.602282073 | 0.995775722 | 1.688660276 |

**STC($^{3}$nσ*/$S_0$)**

| | | | |
|---|---|---|---|
| 6 | -0.443790034 | -1.201173087 | -0.110996007 |
| 6 | -1.779728130 | -1.262635093 | -0.354925023 |
| 6 | -2.496561178 | -0.054272006 | -0.573986042 |
| 6 | -1.860393132 | 1.202651085 | -0.490019037 |

| | | | |
|---|---|---|---|
| 6 | -0.490309037 | 1.239265091 | -0.261591021 |
| 6 | 0.220566015 | 0.048588002 | -0.080095004 |
| 53 | 2.291076165 | 0.134440010 | 0.236414016 |
| 7 | -3.800605275 | -0.124114010 | -0.811117056 |
| 7 | -4.943250358 | 0.172744013 | 1.263942092 |
| 1 | 0.106866007 | -2.116069152 | 0.052454002 |
| 1 | -2.298097167 | -2.211014160 | -0.387093025 |
| 1 | -2.427649177 | 2.111157154 | -0.631976047 |
| 1 | 0.012124001 | 2.194332160 | -0.228343015 |
| 1 | -4.491406321 | -0.764527052 | -1.198709085 |
| 1 | -5.948920430 | 0.237747019 | 1.206954084 |
| 1 | -4.751390341 | -0.601226044 | 1.883765134 |
| 1 | -4.639360332 | 1.003264072 | 1.752017128 |

$^{1}$**2** ($n^2$)

| | | | |
|---|---|---|---|
| C | -0.482623000 | -1.191649000 | -0.084292000 |
| C | -1.791814000 | -1.242724000 | -0.454117000 |
| C | -2.521749000 | -0.022456000 | -0.785060000 |
| C | -1.780122000 | 1.236928000 | -0.784029000 |
| C | -0.458365000 | 1.274803000 | -0.448191000 |
| C | 0.179457000 | 0.071509000 | -0.039644000 |
| H | 0.055417000 | -2.087598000 | 0.191806000 |
| H | -2.317832000 | -2.189309000 | -0.475740000 |
| H | -2.319768000 | 2.137679000 | -1.038629000 |
| H | 0.084919000 | 2.209326000 | -0.428199000 |
| I | 2.112080000 | 0.142552000 | 0.653360000 |
| N | -3.774551000 | 0.023657000 | -1.080870000 |
| H | -4.204705000 | -0.894489000 | -1.048393000 |
| H | -6.011083000 | 0.131514000 | 1.334875000 |
| H | -5.218693000 | -0.507699000 | 2.580617000 |
| H | -4.767827000 | 0.915504000 | 1.977983000 |
| N | -5.084083000 | -0.006098000 | 1.713173000 |

**CI($^1\pi\sigma^*$/$^1n\sigma^*$)**

| | | | |
|---|---|---|---|
| 6 | -0.407167031 | -1.210017089 | -0.101944007 |
| 6 | -1.834400130 | -1.266141094 | -0.256466018 |
| 6 | -2.621398187 | -0.073715005 | -0.417379030 |
| 6 | -1.922509137 | 1.178797087 | -0.358015027 |
| 6 | -0.494431037 | 1.234463088 | -0.171992015 |
| 6 | 0.262140021 | 0.037702000 | -0.057410006 |
| 53 | 2.341267166 | 0.124813011 | 0.176516014 |
| 7 | -3.951261286 | -0.073550007 | -0.592287044 |
| 7 | -4.787910345 | 0.171579013 | 0.959594069 |
| 1 | 0.138547012 | -2.137802155 | -0.014687002 |
| 1 | -2.323704165 | -2.231249163 | -0.281455022 |
| 1 | -2.480542178 | 2.099989149 | -0.460109034 |
| 1 | -0.017984999 | 2.203472157 | -0.139175008 |
| 1 | -4.316060312 | -0.999181073 | -0.753262054 |
| 1 | -5.775080417 | 0.117669007 | 0.751336055 |
| 1 | -4.539901325 | -0.523076036 | 1.652130118 |
| 1 | -4.555817326 | 1.098549077 | 1.284040091 |

**CI($^1n\sigma^*$/$S_0$)**

| | | | |
|---|---|---|---|
| 6 | -0.208177017 | -1.269524092 | -0.349199024 |
| 6 | -1.538077111 | -1.444181102 | -0.535308037 |
| 6 | -2.426041176 | -0.300488020 | -0.491659037 |
| 6 | -1.891076136 | 1.020772076 | -0.214070015 |
| 6 | -0.542376041 | 1.173000082 | -0.040163003 |
| 6 | 0.307036022 | 0.028713004 | -0.100913006 |
| 53 | 2.306645168 | 0.268568022 | 0.181568011 |
| 7 | -3.692212264 | -0.415724028 | -0.665477047 |
| 7 | -5.131747369 | 0.592515040 | 1.066607075 |
| 1 | 0.453833035 | -2.121367153 | -0.387467027 |
| 1 | -1.950167142 | -2.425805174 | -0.721037054 |
| 1 | -2.562553185 | 1.864936134 | -0.177033015 |
| 1 | -0.128742009 | 2.154307156 | 0.139232011 |

| | | | |
|---|---|---|---|
| 1 | -4.276093306 | -0.952402068 | -1.291245093 |
| 1 | -6.124360442 | 0.560942039 | 0.888759064 |
| 1 | -4.984161358 | 0.012485001 | 1.878442135 |
| 1 | -4.944800355 | 1.536440112 | 1.369711099 |

**$S_{N\Sigma}$($^1$nσ*)-Min**

| | | | |
|---|---|---|---|
| 6 | -0.105761005 | -1.244454087 | -0.527098038 |
| 6 | -1.434430101 | -1.465234106 | -0.630774047 |
| 6 | -2.373819171 | -0.370888025 | -0.393872029 |
| 6 | -1.854198133 | 0.935037069 | -0.021993003 |
| 6 | -0.507655039 | 1.113827080 | 0.067229006 |
| 6 | 0.393092027 | 0.031089005 | -0.184288014 |
| 53 | 2.421562172 | 0.334784024 | -0.035432005 |
| 7 | -3.630278263 | -0.602081041 | -0.487595035 |
| 7 | -5.300823379 | 0.772183057 | 0.986048073 |
| 1 | 0.579879044 | -2.060606148 | -0.704828048 |
| 1 | -1.827922133 | -2.437974176 | -0.885666065 |
| 1 | -2.536147182 | 1.746097127 | 0.156640011 |
| 1 | -0.116763008 | 2.086723151 | 0.327983022 |
| 1 | -4.305349309 | -1.252552092 | -0.877249062 |
| 1 | -6.245586451 | 0.751222055 | 0.628190043 |
| 1 | -5.321990384 | 0.240417020 | 1.845514133 |
| 1 | -5.138207369 | 1.729335126 | 1.265241090 |

Cartesian coordinates, total energies, and vibrational zero-point energies for the optimized geometry from the um062x/lanl2dz calculations for the compounds and intermediates considered in this paper are given:

Radical cation **4**

Charge = 1 Multiplicity = 2

| | | | |
|---|---|---|---|
| C | 2.96574000 | 0.00014300 | 0.00002900 |
| C | 2.24242200 | 1.24510700 | 0.00049900 |
| C | 0.86305300 | 1.23643200 | 0.00067900 |
| C | 0.15727200 | 0.00000300 | 0.00080900 |
| C | 0.86302100 | -1.23643600 | 0.00067000 |
| C | 2.24237500 | -1.24492600 | 0.00049000 |
| I | -1.92541700 | -0.00000900 | -0.00021500 |
| N | 4.30734000 | -0.00015400 | -0.00114000 |
| H | 0.31880800 | -2.17292800 | 0.00080300 |
| H | 2.78675300 | -2.18258000 | 0.00056300 |
| H | 2.78667900 | 2.18284200 | 0.00084000 |
| H | 0.31879800 | 2.17289500 | 0.00088500 |
| H | 4.84099300 | 0.86580800 | -0.00192800 |
| H | 4.84041600 | -0.86640700 | -0.00087300 |

Zero-point correction= 0.108931 (Hartree/Particle)

Sum of electronic and thermal Free Energies= -297.902103 Hartree

Radical cation **6**

Charge = 1 Multiplicity = 2

| | | | |
|---|---|---|---|
| C | -2.05412100 | 0.01511200 | -1.26301900 |
| C | -0.83442300 | 0.59260900 | -1.26588900 |
| C | -0.07475500 | 0.85417500 | 0.00004400 |
| C | -0.83438300 | 0.59240900 | 1.26598000 |
| C | -2.05411000 | 0.01496200 | 1.26304500 |
| C | -2.70906400 | -0.31809900 | 0.00000000 |
| N | -3.88702600 | -0.89794700 | -0.00005300 |
| I | 1.66236900 | -0.50965200 | -0.00002600 |
| N | 0.46540300 | 2.27025700 | 0.00008500 |
| H | -2.56707800 | -0.19480800 | -2.19362800 |
| H | -0.35081400 | 0.84964500 | -2.20350500 |
| H | -0.35071700 | 0.84929800 | 2.20360900 |
| H | -2.56708900 | -0.19500600 | 2.19363100 |
| H | -4.37027000 | -1.13832600 | -0.86425500 |
| H | -4.37033000 | -1.13844100 | 0.86408000 |
| H | 1.04851000 | 2.45623800 | 0.83086800 |
| H | 1.04866900 | 2.45621000 | -0.83059700 |
| H | -0.30994400 | 2.95355400 | 0.00000500 |

Zero-point correction= 0.153886 (Hartree/Particle)

Sum of electronic and thermal Free Energies= -354.216392 Hartree
